# DNA Methylation as Drug Sensitivity Marker in RCC: A Systematic Review

**DOI:** 10.3390/epigenomes8030028

**Published:** 2024-07-15

**Authors:** Antonios Koudonas, Georgios Dimitriadis, Anastasios Anastasiadis, Maria Papaioannou

**Affiliations:** 1 First Department of Urology, School of Medicine, Faculty of Health Sciences, Aristotle University of Thessaloniki, 541 24 Thessaloniki, Greece; c3dw9@windowslive.com (A.K.); gdimit@auth.gr (G.D.); dranastasiadis@yahoo.gr (A.A.); 2 Laboratory of Biological Chemistry, School of Medicine, Faculty of Health Sciences, Aristotle University of Thessaloniki, 541 24 Thessaloniki, Greece; 3 Department of Urology, 424 Military Hospital, 564 29 Thessaloniki, Greece

**Keywords:** renal cell cancer, DNA methylation, drug sensitivity, predictive biomarkers

## Abstract

Patient response after treatment of renal cell cancer (RCC) with systemic agents, which include various drug categories, is generally poor and unpredictable. In this context, the ideal drug administration includes tools to predict the sensitivity of the disease to therapy. The aim of this study was to systematically summarize the reports on the predictive value of the methylation status in the systemic therapy of RCC. Only original articles reporting on the association of promoter methylation with the response of patients or cell lines to systemic agents were included in this review. We applied PRISMA recommendations to the structure and methodology of this systematic review. Our literature search concluded with 31 articles conducted on RCC cell lines and patient tissues. The majority of the studies demonstrated a methylation-dependent response to systemic agents. This correlation suggests that the methylation pattern can be used as a predictive tool in the management of RCC with various classes of systemic agents. However, although methylation biomarkers show promise for predicting response, the evidence of such correlation is still weak. More studies on the gene methylation pattern in patients under systemic therapy and its correlation with different degrees of response are needed.

## 1. Introduction

Renal cell cancer (RCC) is one of the most common urological cancers, accounting for 2.2% (431,288 cases) of the total new cancer cases worldwide in 2020. The percentage of cancer-related deaths attributed to RCC is 1.8% [1]. The incidence of RCC varies across regions, with higher rates observed in Europe and North America, among older patients, and in men [2]. Treatment options for localized RCC include the surgical removal of the tumor-bearing kidney, selective surgical excision of the tumor (partial nephrectomy), or destruction of the tumor tissue using thermal ablation techniques [3]. In contrast, locally advanced or metastatic RCC is primarily managed with systemic treatments that prolong life, such as antiangiogenic factors and immunotherapy [3]. RCC is highly resistant to chemotherapy and radiotherapy, but immune checkpoint inhibitors (ICI) have shown promising results in terms of response rates and prolonged time to disease recurrence or cancer-related death, with some patients achieving a complete response [4,5]. However, acquired resistance to treatment often leads to disease recurrence, requiring the use of second-line systemic agents. This cycle of acquired resistance, disease recurrence, and subsequent treatment options is known as therapy sequencing [3].

Given that the success of both first-line therapy and therapy sequencing depends on the biological resistance of RCC, it is reasonable to explore methods for predicting resistance patterns. Pharmacoepigenetics, a subdiscipline of epigenetics, offers a potential strategy for maximizing therapeutic outcomes and avoiding side effects from ineffective systemic agents. Pharmacoepigenetics focuses on studying epigenetic factors that influence drug response and identifying new pharmacologic targets and biomarkers with prognostic or predictive value [6]. Both genetic and epigenetic factors contribute to different responses to systemic therapies, and evaluating both can help develop personalized protocols that adhere to the principles of precision medicine, maximizing therapeutic effects and minimizing side effects [7].

In recent years, a deeper understanding of RCC molecular biology has led to the introduction of new targeted agents and the identification of molecular pathways that may play a role in RCC’s response to systemic therapies. Several molecular factors involved in these pathways are potential predictive biomarkers. Epigenetic alterations, which have also been linked to renal carcinogenesis and tumor aggressiveness [8], offer a promising molecular mechanism with predictive value in the metastatic setting of RCC. Certain genes appear to influence the response to systemic agents in a methylation-dependent manner [9]. Moreover, the frequency of methylation pattern modifications is 2–5 times higher than that of gene mutations [10], suggesting that epigenetic alterations have a stronger effect than mutations in the development of therapy resistance. However, the evidence for the use of predictive biomarkers in clinical practice is inconclusive. The absence of clinically relevant predictive biomarkers is a complex issue [11], indicating that the introduction of such biomarkers into clinical practice may be more challenging than initially anticipated.

The objective of this article is to present the results of a systematic review on the contribution of DNA methylation studies in patient tissues or RCC cell lines to the discovery of predictive biomarkers in RCC and the potential for guiding systemic therapy selection in these patients.

## 2. Results

### 2.1. Study Selection

**Figure 1:**
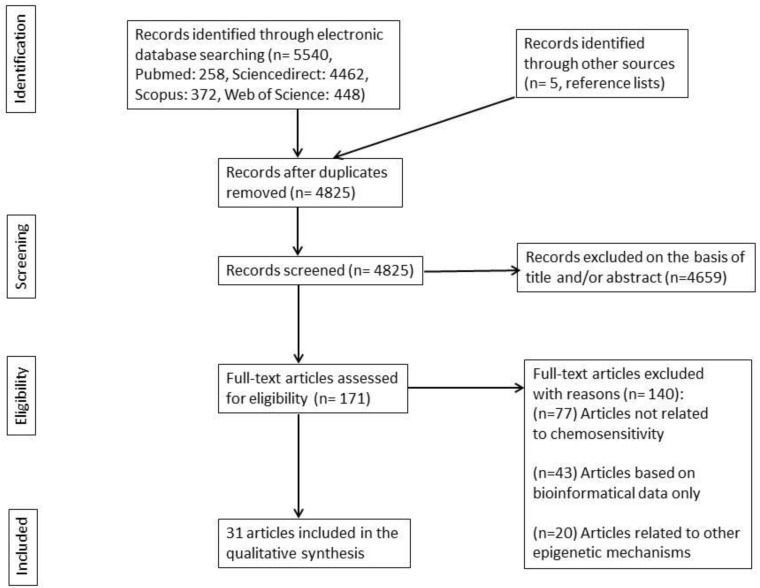
Flow diagram of the literature search.

The search of PubMed, Web of Science, Scopus, and ScienceDirect, along with articles from other sources, provided a total of 5540 articles. After removing duplicates, there were 4,825 articles remaining. The evaluation process began with the appraisal of titles and/or abstracts, resulting in the exclusion of 4,659 irrelevant articles. This left 171 articles for full-text evaluation. After evaluating the full text, 140 articles were excluded for not meeting the eligibility criteria. The remaining 31 articles were included in the present systematic review. The flow diagram of the systematic review is depicted in Figure 1 (the distribution of included articles per electronic database is depicted in Supplementary Table S1).

A summary of the 31 selected articles and their basic extracted data can be found in Table 1, where studies are organized chronologically. In this table, we used both the recent nomenclature of the investigated genes and the gene names as stated in the included studies [12].

### 2.2. Study Characteristics

All included studies were published between 2006 and 2023, indicating that interest in uncovering possible correlations between promoter methylation and drug sensitivity of RCC emerged several years ago and remains strong today. Seven articles reported results only from RCC cell lines; nine articles reported outcomes from patient tissues, and 15 studies included both methodologies (Table 1).

The in vitro studies conducted with RCC cell lines revealed the correlations between the gene methylation status and sensitivity to specified systemic agents. Various molecular methods, such as gene expression analysis (reverse transcription polymerase chain reaction, RT-PCR), protein expression analysis (Western blot), protein identification assays (immunohistochemistry, IHC), methylation status analysis (methylation-specific PCR or MSP and pyrosequencing), and mutation analysis (PCR) were used to establish the relationship between the methylation and gene function. Additionally, several studies applied treatment with demethylating agents (DNA methyltransferase and DNMT inhibition) to induce sensitivity changes through gene demethylation. Functional tests on the same RCC cell lines included DNMT depletion (transfection with antisense DNMT RNA), gene knocking down with short hairpin RNA (shRNA) or small interfering RNA (siRNA), and enforced expression with transfection with gene vectors. These experiments aimed to correlate the gene functional status (inactivation or enforced expression) with the sensitivity in a similar way to the methylation status, which also allows or represses gene function, resulting in changes in sensitivity. In general, in vitro studies aimed for either a direct connection of the methylation status to the sensitivity or an indirect connection through gene expression changes, an effect that can be methylation—driven by or a result of gene manipulations in combination with gene-function-dependent sensitivity. Supplementary Figure S1 depicts both approaches in the form of the Population, Intervention, Comparison, Outcomes, and Study (PICOS) framework. The extracted data from the in vitro studies are summarized in Supplementary Table S2.

The majority of tissue studies had a cohort design, with 10 cross-sectional studies and two case-control studies (Table 1). Many of these tissue studies were combined with results from RCC cell lines to clarify the underlying association of the methylation status of a specified gene with its sensitivity to a specified agent. Researchers suggested that the epigenetic state of a gene could predict the therapy response if the gene was found to be methylated in patient tissues, and the cell line experiments showed different responses to systemic therapy based on different epigenetic states. In four studies [21,31,36,38], researchers retrieved methylation status data for RCC tissues from The Cancer Genome Atlas (TCGA) databank. A more direct approach included the comparison of survival curves, progression-free survival (PFS), and the OS of patients under systemic therapy who had differently methylated tumor tissues in nephrectomy specimens. In one study [26], researchers looked into the possible correlation between epigenetic changes in paired tumor tissues (before and after systemic therapy) and the response to systemic agents. Supplementary Figure S2 depicts the sequence of demonstrating the correlation of promoter methylation to drug sensitivity in any design of included tissue study using the PICOS framework. The extracted data from the tissue studies are summarized in Supplementary Table S3.

### 2.3. Risk of Bias within Studies

The risk of bias assessment for cell line studies did not show significant subjectivity issues. More precisely, critical elements of the methodology, such as the concealment of allocation to groups, experimental conditions, concealment of group characteristics, exposure characterizations, and outcome assessments, were mainly unproblematic or showed minimal flaws concerning bias risk. On the contrary, the assessment of randomization of exposure and completeness of outcome recording and reports demonstrated subjectivity issues, which were detected in seven [16,27,31,32,34,36,38] and three studies [14,15,16], respectively. The majority of studies had up to one item positive for bias risk, while one study [14] had two positive items, and two studies [15,16] had three and four positive items out of nine items. Supplementary Table S4 presents analytical data regarding bias assessment in cell line studies.

Regarding tissue studies, bias risk assessment revealed important issues relating to the objectivity of the formation of the comparing groups and their comparability in terms of confounding factors that can also affect the outcomes. Several studies [32,34,36,41,43] demonstrated significant flaws in the selection process with subsequent suboptimal representativeness of the groups, which resulted in a two-point loss in the study rating. Other studies compared groups uncontrolled for important factors like age, sex, or others [19,20,21,22,23,26,29,37,39,40], which also resulted in a two-point loss in the comparability rating. In the outcome/exposure ascertainment field, several studies [16,23,25,27,33,38,41,43] showed bias elements, which reduced their respective ratings by one point. Supplementary Table S5 depicts analytical data regarding bias assessment in tissue studies.

### 2.4. Results from Studies on Cell Lines

#### 2.4.1. ATP Binding Cassette Subfamily G Member 2 (*ABCG2*)

In 2006, To et al. tested three RCC cell lines in terms of the methylation status of the promoter of the *ABCG2* gene and the respective gene expression [13]. The UOK121 and UOK143 cell lines were found hypermethylated at the promoter of the *ABCG2* gene, and the gene expression was downregulated. On the other hand, the *ABCG2* expression was normal in UOK181 cells in correlation with its unmethylated promoter. The expression differences were abolished after treatment with decitabine. The cytotoxicity assay showed that the methylated UOK121 and UOK143 cell lines were significantly more sensitive than the unmethylated UOK181 under treatment with any of the *ABCG2* substrate drugs. *ABCG2* inhibition with fumitremorgin C (FTC) pretreatment reduced the half-maximal inhibitory concentration (IC_50_) of UOK 181 under any of the previous drugs by 66%, while the respective sensitivities of UOK121 and UOK143 under the same conditions showed only a limited effect of FTC.

#### 2.4.2. Ras Association Domain Family Member 1 (*RASSF1*)

The *RASSF1* gene was found to be silenced due to its promoter hypermethylation in 90–100% of RCC specimens. To investigate if the resistance to the interferons (IFNs)-induced apoptosis was due to *RASSF1* hypermethylation, Reu et al. tested the effect of IFNs after preventing DNA hypermethylation, either with a decitabine pretreatment or DNMT1 depletion [14]. Initially, resistant RCC cell lines showed increased apoptosis after treatment with decitabine or DNMT depletion, while *RASSF1* was re-expressed after treatment. The enforced expression of *RASSF1* by a lentiviral infection resulted in the same effect. On the contrary, inhibition of *RASSF1* by siRNA decreased the apoptotic effect of IFN.

#### 2.4.3. XIAP-Associated Factor 1 (*XAF1*)

In 2006, Reu et al. assessed the dependence of IFN-induced apoptosis on the *XAF1* gene methylation status by applying DNMT inhibition (treatment with decitabine) or DNMT depletion (transfection with antisense RNA) on the RCC cell lines ACHN and SK-RC-45 [15]. The pretreatment resulted in an increase in apoptotic cells by up to 85% under IFN treatment, while RCC cell lines without pretreatment were resistant to IFNs (<10% TdT-mediated dUTP-biotin nick end labeling, TUNEL+ cells). Simultaneous treatment of ACHN cells with *XAF* siRNA and decitabine resulted in *XAF1* knocking down and reduced apoptosis under IFN β (18%), compared to cells treated only with decitabine plus IFN β (58%). Enforced *XAF1* expression through a plasmid vector on DNMT-depleted ACHN cells resulted in a low apoptosis ratio (6.5%) under a low IFN dose, while treatment with 500 U/mL IFN-β apoptosis reached up to 80%. Empty vector-carrying cells were resistant even to high IFN β doses.

#### 2.4.4. *p73*, a Homolog of *p53*

In 2007, the National Cancer Institute drug-screening panel (NCI-60 panel), which includes seven renal cancer cell lines, was used by Shen et al. to determine the methylation status across 32 promoter-associated CpG islands and to correlate the methylation status with the response to systemic agents [17]. Two renal cancer cell lines, TK10 and 786-O, were found to have different responses to cisplatin. The increased sensitivity to cisplatin treatment of the 786-O cell line was correlated with the hypermethylation of the *p73* gene promoter and the minor expression of the gene. On the other hand, the cisplatin-resistant TK10 cell line expressed *p73*, and the promoter of *p73* was unmethylated. Experimentally, *p73* knocking down by siRNA downregulated the expression of p73 and increased the sensitivity to cisplatin treatment.

#### 2.4.5. Connexin 32 (*Cx32*)

In 2010, Takano et al. found that *Cx32*, a tumor suppressor gene, was frequently silent in RCC due to the hypermethylation of its promoter [18]. The pretreatment of Caki-1, a representative RCC cell line, with decitabine, resulted in the restoration of *Cx32* gene expression. *Cx32* repressed the expression of P-glycoprotein (*P-gp*) and, therefore, enhanced the vinblastine (VBL)-induced cytotoxicity.

#### 2.4.6. DNA Mismatch Repair Gene (*MSH2*)

Ponnusamy et al. developed the Caki-1^LA^ and Caki-1^HA^ renal cancer cells as a model for the low (LA) and high adoption (HA) to chronic exposure to oxidative stress [24]. In particular, Caki-1 cells were exposed for six months to H_2_O_2_. The cells were adapted to chronic oxidative stress and had developed an enhanced resistance to doxorubicin and cisplatin. The oxidative-stress-induced resistance to the chemotherapeutics was correlated with the decreased expression of the *MSH2* gene. DNA pyrosequencing revealed hypermethylation in one of the six CpG sites in the promoter region of the *MSH2* gene, thus suggesting the possible role of oxidative-stress-induced DNA hypermethylation in silencing the *MSH2* gene. Subsequently, treatment with decitabine resulted in the restoration of *MSH2* gene expression and enhanced sensitivity to doxorubicin-induced cytotoxicity.

#### 2.4.7. Schlafen 11 (*SLFN11*)

In 2016, the NCI-60 panel was used by Nogales et al. to unveil the correlation of CpG methylation with the response to the platinum-derived chemotherapeutic drugs cisplatin and carboplatin [28]. TK10 and A498 RCC cell lines were found to have the CpG island region of the *SLFN11* gene hypermethylated. The absence of *SLFN11* protein expression, a putative DNA/RNA helicase, was associated with reduced sensitivity to the above chemotherapeutic compounds. In general, the methylation status of the *SLFN1* gene promoter across all cell lines proved to be predictive of the response to the platinum drugs.

### 2.5. Results from Studies on Cell Lines and Tissues

#### 2.5.1. XIAP-Associated Factor 1 (*XAF1*)

In 2006, Lee et al. investigated the relationship between the *XAF1* promoter gene methylation and protein expression within urogenital malignancies [16]. In total, 10 of the 15 RCC cell lines showed very low or no *XAF1* protein expression. The MSP analysis showed that the lack of protein expression was due to the gene promoter methylation. On the contrary, the rest of the cell lines with normal *XAF1* protein expression showed no promoter methylation. Moreover, a significant reduction in *XAF1* expression was revealed in 7 out of 20 RCC samples, and six out of these seven samples showed increased gene promoter methylation. On the other hand, no methylation was detected in the sum of noncancerous and normal *XAF1* protein-expressing RCC tissues. Transfection assays with the 253J and HT1376 bladder cancer cell lines were performed to elucidate the relation between *XAF1* gene expression and cell chemosensitivity. The ectopic overexpression of the *XAF1* protein in the 253J cell line, which lacks endogenous *XAF1* protein expression, significantly increased the apoptotic response to chemotherapeutic drugs, such as etoposide and 5-fluorouracil (5-FU) (*p* < 0.01). On the contrary, the HT1376 cells showed an attenuated response to chemotherapeutics after targeted inhibition of *XAF1* protein expression with siRNA.

#### 2.5.2. Neurofilament Heavy Chain (*NEFH*)

Using the quantitative methylation-specific PCR (qMSP), Dubrowinskaja et al. found that the *NEFH* promoter was methylated both in RCC cell lines, with relative methylation >25% in two of six cell lines and in cancerous tissues compared to the paired normal specimens [19]. A survival analysis showed that *NEFH* methylation was a significant prognostic factor (a significantly shortened PFS was recorded in patients with relative methylation higher than 5.9%, *p* < 0.001, hazard ratio (HR) = 8.61 [3.03–24.5, 95%CI]). Patients undergoing antiangiogenic therapy showed OS depending on the methylation status (29.8 vs. 9.8 months for patients with low and high methylation, respectively, *p* = 0.028). By using a cut-off of 6 months for PFS, *NEFH* methylation was correlated with therapy failure with a sensitivity of 0.91 [0.62–0.98. 95%CI].

#### 2.5.3. Fms-Related Receptor Tyrosine Kinase 1 (*FLT1*), Kinase Insert Domain Receptor (*KDR*)

In 2015, Kim et al. investigated the effect of the methylation status of *FLT1* and *KDR* genes, which express vascular endothelial growth factor (VEGF) receptors 1 and 2, respectively, on the efficacy of drugs acting on the VEGF pathway, namely, bevacizumab (monoclonal antibody against VEGF-A), axinitinib, and sunitinib (tyrosine kinase inhibitors, TKIs, both blocking the signaling pathways of VEGF receptors). The researchers performed in vitro experiments, which showed that growth inhibition induced by bevacizumab or anti-*KDR* antibodies was not dependent on the methylation of *FLT1* or *KDR* genes [25]. On the contrary, cell lines with *FLT1* hypermethylation were more resistant to anti-*FLT* peptides, sunitinib, and axitinib. Pretreatment with decitabine increased the effect of sunitinib and axitinib on *FLT1* hypermethylated cell lines. Only *FLT1* knocking down decreased the effect of anti-*FLT1* peptides, sunitinib, and axitinib on respective cell line models. The methylation status of normal adjacent tissue was found to be significantly different compared to cancerous tissue for both genes. Responders to sunitinib showed significantly lower *FLT1* methylation compared to non-responders. The results of the study showed that hypermethylation of *FLT1*, but not *KDR*, affects the efficacy of both TKIs in RCC.

#### 2.5.4. Apoptosis-Associated Speck-like Protein Containing a Card/Target of Methylation-Induced Silencing 1 (*ASC/TMS1*)

In 2015, Liu et al. assessed the pro-apoptotic gene *ASC/TMS1* for its methylation status in RCC cell lines and tumor/normal tissues [27]. All cell lines were methylated, while *ASC/TMS1* was downregulated or silenced. Tumor-specific methylation of *ASC/TMS1* was demonstrated from the methylation percentage in tumor tissues (83/202, 41.1%) compared to normal tissues (3/25, 12%). Pretreatment with decitabine or enforced *ASC/TMS1* expression potentiated cell death of initially *ASC/TMS1*-silenced 786-O cells after treatment with a chemotherapeutic agent etoposide or doxorubicin. Knocking down the *ASC/TMS1* gene on Caki-2 cells induced an attenuated *p53* activation after treatment with etoposide or doxorubicin.

#### 2.5.5. Organic Cation Transporter 2 (*OCT2*)

According to a study by Liu et al., the *OCT2* gene was hypermethylated in tumor tissues with strong *OCT2* repression (*p* = 0.0001) and in tumor tissues with weak *OCT2* repression (*p* = 0.006) compared to respective non-tumor tissues [30]. The difference in *OCT2* expression was also revealed on the RCC tissue microarray (positive *OCT2* staining in 24 of 31 non-tumor samples, negative in all tumor samples). Pretreatment of RCC cell lines with decitabine increased oxaliplatin cellular accumulation and resulted in a multifold decrease in IC_50._ This effect did not take place in modified RCC cell lines after *OCT2* knocking down. The combination of decitabine with oxaliplatin resulted in the inhibition of rapid tumor growth in xenografts, while monotherapy with either decitabine or oxaliplatin had no effect.

In 2016, Winter et al. assessed the *OCT2* methylation status across discrete CpGs in primary tumor samples, metastases, and cell lines and found no significant differences between primary tumor tissues and metastatic tissues [32]. On the contrary, RCC cell lines had significantly higher methylation levels than primary tumors or metastases. Treatment of Caki-2 cells with decitabine resulted in a four-fold increase in *OCT2* mRNA and a significant increase in induced apoptosis by the addition of cisplatin compared to treatment with cisplatin alone (*p* < 0.01).

#### 2.5.6. Disabled Homolog 2-INTERACTING Protein (*DAB2IP*)

In 2016, Zhou et al. processed TCGA data and found that the *DAB2IP* gene was methylated in 82% of tumor tissues (130/159) after comparison to their matched renal tissues [31]. *DAB2IP* expression was detected in 95% of normal kidney tissues, while it was decreased in 54% of tumor tissues in the patient cohort, suggesting gene silencing in tumor tissues. RCC cell line treatment with decitabine increased *DAB2IP* expression, revealing the association between *DAB2IP* methylation and expression. RCC cell lines with dysfunctional *DAB2IP* (knocked down 786-O cells, 786-O KD and Sut002 vector control, Sut002VC) showed higher resistance to temsirolimus compared to RCC cell lines with relatively functional *DAB2IP* (786-O and *DAB2IP*-expressing Sut002 cells, Sut002DAB2IP). Tumor enlargement in xenografts under treatment with temsirolimus was faster in cases of inoculation with 786-OKD and Sut002VC. Survival curves of a small patient cohort treated with a mammalian target of rapamycin (mTOR) inhibitors revealed a decreased median survival (23.6 vs. 46 months, *p* = 0.41) for patients with low *DAB2IP* expression (data from TCGA).

#### 2.5.7. Apoptosis Stimulating Protein of p53 1 (*ASPP1*)

According to a study by Wang et al., in vitro experiments showed that *ASPP1* hypermethylation at the promoter region suppressed the *ASPP1* expression, while the latter was restored after treatment of RCC cells with decitabine [34]. At the patient level, *ASPP1* expression was found to be downregulated by 3.9-fold in the mRNA transcript level and 4.9-fold in the protein level in RCC tissues compared to paired normal controls (*p* < 0.01). The influence of *ASPP1*’s functional status in response to chemotherapy was tested through 5-FU treatment of cell lines after enforced *ASPP1* expression, which showed higher sensitivity compared to cell lines with non-functional *ASPP1*.

#### 2.5.8. Leukemia Inhibitory Factor Receptor (*LIFR*)

In 2018, Lei et al. found that the *LIFR* protein level was significantly lower in 25 tumor samples compared to normal matched samples (*p* < 0.001), while transcriptomic data from TCGA showed that *LIFR* mRNA levels were significantly lower in tumor samples than in normal samples (*p* < 0.001) [36]. Methylation data analysis from TCGA revealed hypermethylation in RCC samples (*p* < 0.001). A significant negative correlation between methylation and *LIFR* transcription resulted in three *LIFR* gene sites (cg03864479: r = −0.435, *p* < 0.001, cg06182018: r = −0.379, *p* < 0.001, cg06182018: r = −0.140, *p* = 0.012). *LIFR* knockdown on Caki-2 cells increased sensitivity to verteporfin (IC_50_ value = 10.38 vs. 16.91 μmol for control cell line), while a correlation analysis between drug sensitivity and *LIFR* mRNA levels on nine RCC cell lines in the CCLE database revealed a strong correlation for two drugs (PHA-665752 with r = 0.707, *p* = 0.033, PF2341066 with r = 0.707, *p* = 0.033).

#### 2.5.9. Doublecortin-like Kinase 1 (*DCLK1*)

Weygant et al. performed a methylation data analysis of 159 paired RCC plus normal adjacent tissues from TCGA–KIRC, which showed a strong *DCLK1* promoter hypomethylation that could perform as a diagnostic biomarker in distinguishing cancerous tissue (Area Under Curve, AUC = 0.838 ± 0.024 for β-promoter) [21]. *DCLK1* overexpression was also revealed by immunochemistry in stage 2–3 vs. stage 1/normal tissues (*p* < 0.002). *DCLK1* knocking down by treatment of Caki-2 cells with *DCLK1* siRNA reduced viability by 30% in proliferation assay under sunitinib therapy.

#### 2.5.10. Paraoxonase 1 (*PON1*)

According to a study by Li et al., methylation data analysis of the TCGA databank showed significant hypermethylation of the *PON1* gene in RCC tissues [38]. The same result was found in the methylation study of 15 fresh frozen RCC samples compared to matched adjacent renal tissue specimens, with 12 of 15 RCC tissues found hypermethylated for the *PON1* gene. The effect of DNMT inhibition (decitabine) on the sensitivity of RCC cell lines with proven hypermethylation to sunitinib suggests a possible connection of the *PON1* methylation status to the sensitivity of RCC to sunitinib.

#### 2.5.11. Glutaminyl-Peptide Cyclotransferase (*QPCT*)

In 2019, a study by Zhao et al. showed that the treatment of RCC cells with decitabine could induce an intense *QPCT* expression through modification of the methylation status [39]. Increased *QPCT* expression was associated with reduced sensitivity to sunitinib, while *QPCT* suppression with siRNA resulted in reduced viability after sunitinib treatment. Patients considered as responders to sunitinib had tumors with higher *QPCT* methylation, while an adjuvant treatment with sunitinib was associated with a significant improvement in PFS only in the patient subgroup with low *QPCT* expression. Interestingly, the *QPCT* protein level was higher in the plasma of non-responsive patients.

#### 2.5.12. Transposable Elements (TE)

Transposable elements are mainly endogenous retroviruses, which have been integrated into the human genome and are normally repressed via epigenetic mechanisms. Activated TE in cancer cells induces viral mimicry and, consequently, an immune response. In this study by de Cubas et al., TE expression was modulated via the hypomethylating effect of decitabine on RCC cell lines [40]. Activated TE expression triggered antiviral signaling through the upregulation of pattern recognition receptors (PRR). Expression of corresponding genes (*DDX58*, *IFIH1*, *DHX58*) was upregulated in RCC tissues of a group of patients with good response to Programmed cell Death1/Programmed cell Death-Ligand 1 (PD1/PD-L1) inhibitors in contrast to non-responders (*p*: 0.006, *p*: 0.011, *p*: 0.027).

#### 2.5.13. Ubiquinol Cytochrome c Reductase Hinge (*UQCRH*)

The *UQCRH* gene controls the production of a mitochondrial complex III component, which is functionally associated with the Warburg effect and has a putative role in the induction of apoptotic cell death. In the study by Miyakuni et al., immunohistochemical analysis showed that the *UQCRH* expression in RCC tissues was lower compared to normal adjacent tissues, regardless of the malignity grade [41]. Methylation analysis results showed that the highly malignant OS5K cell line was methylated, which was in line with methylation data from a database analysis relating to RCC cells. Everolimus treatment induced more intense apoptosis in the less-methylated OS-RC-2 cells than in OS5K cells, while the apoptosis of the latter was restored after pretreatment with decitabine. The inoculation of pretreated decitabine OS5K cells in mice was associated with an increased therapeutic effect of everolimus on the primary tumor of the mice.

#### 2.5.14. T-Cell Activation Inhibitor, Mitochondrial (*TCAIM*)

The *TCAIM* gene encodes a mitochondrial protein that associates with mitochondrial calcium uptake and additionally plays a role in T-cell priming capacity and activation. Ye et al. found that the *TCAIM* gene was more methylated in RCC tissues compared to normal adjacent tissues, while the *TCAIM* protein levels of the latter were higher [43]. *TCAIM* functional manipulations in RCC cell lines revealed the importance of the gene functional status to sensitivity under treatment with sunitinib. More precisely, *TCAIM*-enforced expression provoked an augmented sensitivity, while *TCAIM* silencing had the opposite effect. Respective gene manipulation in inoculated RCC tumors in mice affected tumor growth in the same way; namely, gene-enforced expression decelerated tumor growth, while gene silencing had an accelerating effect.

### 2.6. Results from Studies on Tissues

#### 2.6.1. Von Hippel-Lindau (*VHL*)

*VHL* gene status analysis was performed by Choueiri et al. as part of a clinical trial on metastatic RCC (mRCC) patients treated with pazopanib [20]. The methylation status and mutational status of 78 tissue samples were assessed before treatment and did not correlate with the overall response rate (ORR) and PFS. In conclusion, the *VHL* gene status had no predictive value for pazopanib activity.

Data from a prospective study by Motzer et al., which focused on different sunitinib–based treatment schedules of patients with advanced RCC, were analyzed to discover predictive biomarkers relating to patients under sunitinib treatment [23]. *VHL* methylation was detected in 14 out of 132 patients. Methylation prevalence across patient groups with different outcomes (0% in the complete response group, 10% in the partial response group, 8% in the stable disease group, and 17% in the progressive disease group) showed no correlation between the methylation status and patient clinical course.

According to a study by Stewart et al., a methylation status analysis in 14 paired RCC specimens (before and after treatment with sunitinib) showed that the *VHL* promoter region 7896829 was significantly more methylated after sunitinib (14% of patients with hypermethylation before treatment, 64% post-treatment, *p* < 0.001, False Discovery Rate, FDR = 0.077) [26]. The response of each patient did not correlate with the extent of *VHL* hypermethylation (*p* = 0.896), suggesting that *VHL* methylation changes take place due to sunitinib regardless of therapy resistance and disease progression.

In 2019, Kammerer-Jacquet et al. divided a cohort of 90 patients into an LTR (long-term responders) group, with a minimum response of 18 months to sunitinib and to other patient groups [37]. The *VHL* methylation status was assessed in samples taken at diagnosis or during follow-up. Promoter methylation was found in 10 out of 90 cancerous tissues (2 from the LTR group, 8 from other patients). No association was found between the *VHL* methylation status and the response duration to sunitinib (*p* = 0.718).

#### 2.6.2. Cystatin-M and Ladinin 1 (*CST6*, *LAD1*)

In 2014, Peters et al. analyzed primary tumor tissues from 18 patients receiving antiangiogenic therapy in terms of *CST6* and *LAD1* methylation status, and the results were evaluated for their predictive value in a clinical disease course [22]. Hypermethylation was found in 8/18 and 10/18 tumor samples for *LAD1* and *CST6*, respectively. Kaplan–Meier curves revealed a shorter PFS and OS for patients under antiangiogenic therapy with *LAD1* hypermethylation (median PFS = 2 vs. 11.4 months for low methylation, *p* = 0.004, HR = 6.4 [1.6–26, 95% CI] and median OS= 3.4 vs. 16.4 months for low methylation, *p* = 0.043, HR = 2.9 [1.0–8.6, 95% CI] or with *CST6* hypermethylation (median PFS = 2 vs. 11.4 months for low methylation, *p* = 0.009, HR = 4.1 [1.3–12.6, 95% CI] and median OS = 3.4 vs. 22.9 months for low methylation, *p* = 0.011, HR = 4.1 [13.0–13.4, 95% CI]. Using a cut-off of 6 months to distinguish between responders and non-responders, the *LAD1* methylation status predicted therapy failure with specificity = 1.0 [0.65–1.0, 95% CI], sensitivity = 0.727 [0.43–0.90, 95% CI], while the C*ST6* methylation status had specificity = 0.857 (0.49–0.97, 95% CI] and sensitivity = 0.818 (0.52–0.95, 95% CI] in predicting therapy responses.

#### 2.6.3. Genome-Wide Methylation Study for Prediction of Response to Sunitinib

In 2015, Beuselinck et al. performed a transcriptomic analysis in primary tumor tissues of mRCC patients who were treated with sunitinib and followed up for an estimation of the PFS and OS, and they identified four discrete clear cell RCC (ccrcc) molecular subtypes (1–4) with different clinical courses (shorter PFS and OS for ccrcc1/4, *p* = 0.001 and 0.0003 respectively) [29]. The methylation pattern of ccrcc1/4 was characterized by global hypermethylation. Genes related to polycomb targets (PRC2, SUZ12, H3K27m3) were found downregulated by hypermethylation in ccrcc1/4, while genes involved in immune responses and mitotic cycles in ccrcc4 tumors were upregulated by hypomethylation. Methylation status differences were part of the molecular differences, which divided mRCC patients into the above subtypes and were of predictive value in RCC cases treated with sunitinib.

#### 2.6.4. Synaptopodin 2 (*SYNPO2*)

In the study by Pompas-Veganzones et al., the *SYNPO2* methylation status was assessed in 63 cancerous tissue specimens from RCC patients before antiangiogenic therapy [33]. Univariate analyses of patients under therapy showed an association between methylation and PFS (*p* = 0.004, HR = 0.43 [0.24–0.77, 95%CI]), while CSS (*p* = 0.003, HR = 0.30 [0.2–0.72, 95%CI]) and OS (*p* = 0.003, HR = 0.42 [0.23–0.78, 95%CI]) were found increased for methylated cases by a Kaplan–Meier curve analysis. Multivariate Cox analyses corroborated the predictive value of methylated *SYNPO2* as an independent factor for PFS (*p* = 0.009, HR = 0.45 [0.25–0.82, 95%CI]) and disease-specific survival (DSS) (*p* = 0.006, HR = 0.4 [0.2–0.76, 95%CI]), OS (*p* = 0.01, HR = 0.45 [0.25–0.82, 95%CI]) for patients under therapy.

#### 2.6.5. Multiple Genes

In this study, Verbiest et al. applied the molecular classification of RCC into ccrcc1-4 subtypes, which was established experimentally by Beuselinck et al. (above-mentioned study in the results on tissues section of the current manuscript) [29], in a cohort of 28 mRCC patients treated with pazopanib [35]. A class effect of VEGFR-TKIs across ccrcc1-4 subtypes was confirmed, with a PFS of 9 months (mo), 5 mo, and 3 mo (*p*: 0.011) and an OS of 69 mo, 19 mo, and 5 mo (*p*: 0.003) for ccrcc2+3, ccrcc1, and ccrcc4 patients, respectively. Survival differences were confirmed from the tumor volume reduction at the time of best response, with −34%, −6%, and −2% for ccrcc2+3, ccrcc1, and ccrcc4, respectively. The dichotomization of patients into ccrcc2+3 vs. ccrcc1+4 groups provided a significant predictive factor in the bivariate Cox proportional hazards model, both for PFS (*p*: 0.026) and OS (*p*: 0.04).

#### 2.6.6. Cytotoxic T Lymphocyte–Associated protein 4 (*CTLA-4*)

The *CTLA4* gene expresses an immune checkpoint receptor, which downregulates immune responses and comprises a target for a specific ICI class (anti-CTLA4). In 2021, Klümper et al. aimed to investigate the prognostic and predictive value of the *CTLA4* methylation status in RCC patients [42]. A methylation analysis showed a significant hypomethylation in tumor specimens. Survival analyses demonstrated that *CTLA4* hypomethylation can predict a shorter OS in patients without ICI therapy. Interestingly, *CTLA4* hypomethylation predicted a favorable PFS (HR = 1.94 [1.09–3.44, 95% CI], *p* = 0.024) and OS (HR = 2.14 [1.01–4.57, 95% CI], *p* = 0.048) in patients who underwent ICI therapy. The above effect remained statistically significant after adjustment for the International Metastatic RCC Database Consortium (IMDC) risk score.

## 3. Discussion

### 3.1. Summary of Evidence

Systemic therapy is one of the main treatment options for cancer, but resistance and toxicity can occur after the use of these drugs. Drug resistance, whether intrinsic or acquired, is caused by factors such as poor drug accumulation, increased drug exportation, altered drug targets, increased DNA repair, and repression of apoptosis. These changes in cell function may also have a genetic basis, but the high prevalence and reversibility of drug resistance suggest that epigenetic changes may also play a role [44]. Promoter methylation patterns can change reversibly with a frequency 2–5 fold higher than the frequency of gene mutations, which are irreversible [10,45]. The prognostic value of promoter methylation in RCC tissues, as reflected by the increasing number of prognosis-relevant gene epigenetic alterations [46], suggests that it could be a useful predictive marker. These epigenetic alterations can also be detected in normal adjacent tissues [47].

To investigate the role of promoter methylation in drug resistance, we conducted a systematic search of the literature for original articles on the prediction of drug resistance in RCC using epigenetic biomarkers. Our search yielded 31 articles that met the inclusion criteria. Researchers used either cell lines or tissues as material for methylation or functional studies, plus data from patient clinical courses. Several of the genes that demonstrated a putative role as predictive biomarkers are either well-known tumor suppressors (*RASSF1*, *XAF1*, *ASC/TMS1*, *ASPP1*, *DAB2IP*) or DNA damage response regulators (*SLFN11*). In addition to the above genes, other biological pathways showed a putative role in the prediction of response to systemic therapy. These pathways included the VEGF pathway (*FLT1* gene), the regulation of transmembrane molecule transportation (*ABCG2*, *Cx32*, *OCT2* genes), the regulation of an epithelial–mesenchymal balance (*DCLK1*, *CST6*, *LAD1* genes), and the regulation of immunogenic function (TEs, *TCAIM*, *CTLA4* genes). Several studies reported the existence of discrete transcriptomic profiles (multigene classifiers) with different metabolic characteristics among RCC cases, and methylation was one of the most determining factors for transcriptomic differentiation. Interestingly, the *VHL* gene, which comprises a tumor suppressor with a role in RCC carcinogenesis, did not demonstrate methylation alterations with predictive value. Regarding the methodology of the included studies, the researchers applied laboratory assays, statistical methods, and a study design, which were heterogeneous. Moreover, the selection of the systemic agents that were evaluated in the included studies depended on the time period of the respective research protocol; namely, several classical chemotherapeutics and initial forms of immunotherapy were tested in the older reports, while TKIs and the newest immunotherapeutic regiments were evaluated in the most recent studies. Notably, the vast majority of the analyzed cell lines had a clear-cell origin, which suggests that the results apply mostly to the same RCC histology (Supplementary Table S6). Among the analyzed cell lines, only ACHN and SKRC39 (from metastatic lesions of primary papillary RCC), UOK112 and SNU482 (from primary papillary RCC), and two additional cell lines (SN12C, TK-10) represented the non-clear-cell histologies [48]. Regarding the representability of the tissue samples of the included studies for the various RCC histological subcategories, only a minority of the analyzed tissue specimens were related to non-clear-cell RCC cases (Supplementary Table S7). This finding may render the results applicable for the most frequent RCC subtype (clear-cell histology) but is not representative of the non-clear-cell histologies. Moreover, the studies that performed bioinformatical data analysis used the information from the TCGA-KIRC cohort (relating to clear-cell RCC), except from a report by Li et al., which performed an analysis of TCGA-KIRP (relating to papillary RCC) data [38].

Cell line studies aim for direct or indirect evidence of the relationship between gene methylation and sensitivity. Direct evidence came from methylation data obtained from methylation studies or methylation manipulations (DNMT inhibition) combined with sensitivity data from cell lines exposed to chemotherapeutics and with a known methylation status. On the contrary, indirect evidence came from chemosensitivity data combined with gene manipulations (knocking down, enforced expression), given that methylation also affects gene function by repressing its expression. Several studies provided both direct and indirect evidence [13,14,15,17,18,25,27,30]. Among them, studies on *RASSF1* and *XAF1* genes [14,15] were characterized by a moderate risk of bias, with two and three positive items, respectively, while studies on *FLT1*, *KDR*, *ASC/TMS1*, and *OCT2* genes combined outcomes in the experimental part with results from an observational part on tissues [25,27,30]. Five studies provided only direct evidence [24,28,32,38,41] and had no major bias issues, and three of them presented combined results from cell lines and tissues [32,38,41]. Seven studies included only indirect evidence, and all of them corroborated results on cell lines by adding outcomes from tissues [16,21,31,34,36,40,43]. Only one study on the *XAF1* gene [16] was assessed as having significant bias issues. Lastly, one study on the *NEFH* gene [19] provided methylation data but no chemosensitivity data of the cell lines. Methylation studies of this article resulted in unbiased outcomes, yet they did not point to the possible relationship between gene methylation and chemosensitivity.

Regarding reports from tissues, 10 studies had a cross-sectional design. These studies provided outcomes on tissues and experimental data on cell lines, combining methylation prevalence or gene expression data in tissues with methylation- or gene-function-dependent sensitivity of cell lines. Six studies tested the methylation status in samples in the form of fresh frozen tissues, formalin-fixed paraffin-embedded (FFPE), or tissue microarray [16,27,30,32,38,41], while three articles reported results of expression studies on tissues and outcomes of methylation studies from TCGA data [21,31,36]. In one study on the *ASPP1* gene, the researchers found reduced mRNA and protein expression in fresh frozen cancerous tissues and conducted in vitro experiments, which showed that *ASPP1* hypermethylation suppressed *ASPP1* expression, and additionally, chemosensitivity to 5-FU was analogous to *ASPP1* expression [34]. The above findings suggest a putative inverse association of methylation to chemosensitivity. Twelve studies on tissues [19,20,22,23,25,29,31,33,35,39,40,42] had a cohort design and demonstrated the difference in the clinical course of patient groups with different methylation or gene function characteristics. Five cohort studies on *NEFH*, *FLT1*, *KDR*, *DAB2IP*, *QPCT*, and TE genes included outcomes from cell line experiments as corroborating the association of drug sensitivity with gene methylation [19,25,31,39,40]. Almost all cohort studies tested the methylation status by comparing tissue groups in the form of fresh frozen samples or FFPE. Most cohort studies [19,20,22,23,29,39,40] tested were not adjusted for other factors tissue groups, with subsequent low ratings in comparability. Minor bias issues in group formation [19,25,35,39,40] and outcome assessment [23,25,33] were recorded in the bias risk rating. Two studies, which had a case-control design, compared the *VHL* methylation status of patient groups with different outcomes (responders, non-responders) and concluded that there was no association of response to systemic therapy with *VHL* methylation [26,37]. Both studies tested were unadjusted for other factor groups, which resulted in a low comparability rating.

This review is the first attempt to examine the body of evidence on predicting responses to systemic agents in RCC through DNA methylation studies. The discovery of predictive biomarkers based on promoter methylation in RCC is still in its infancy. Nevertheless, this initial evaluation of biomarker candidates is the first step in planning and performing large-scale studies. In the current review, these initial evaluations were based on cell lines and tissue studies.

Cell line studies are useful for investigating the biological properties of biomarker candidates and predicting the reactions to treatments. They can be considered the first step in biomarker discovery because their results are necessary for further designing and conducting multi-institutional validation studies. The cost-effectiveness and simplicity of epigenetic manipulation and molecular characterization are additional advantages. However, associating the gene methylation status with chemosensitivity requires several steps and assumptions. In the included studies with in vitro experiments, researchers mostly manipulated the cell line methylome through decitabine treatment and measured the alteration of cell chemosensitivity, a process that does not exclude the effect of other genes on the measured outcome (chemosensitivity). To further emphasize the role of the gene of interest, most studies included additional functional tests, which demonstrated the association of gene function (enhanced by gene vectors, silenced by siRNA/shRNA) with cell chemosensitivity. Since methylation manipulation induces similar gene function alterations, the researchers extrapolated that the methylation status could affect the gene of interest-dependent chemosensitivity. The above logical sequence distinguishes the gene of interest as the important determinant of the drug effect in cancer cells and compensates for the lack of applied targeted methylation manipulation methods on a specific genetic locus (epigenetic editing of DNA methylation) in the included studies. Another drawback of in vitro experiments is that the epigenetic signature of cell lines may not reflect epigenetic profiles found in RCC cases. According to a study [32], RCC cell lines displayed not only genome-wide hypermethylation compared to primary tumors or metastases but also altered DNA methylation of drug targets and other pharmacogenes. These data are in line with corresponding data of other tumor entities (breast, prostate, colon, etc.) [49], which makes usage of RCC cell lines for epigenetic studies on predicting therapy response questionable.

Tissue samples seem to provide more direct evidence for the discovery of predictive epigenetic biomarkers. However, tissue sampling and clinical data registration are strenuous operations that require the strict implementation of legal precautions and standardized procedures in the sample acquisition, storage, and analysis. According to a study, sampling procedures and hypoxia/ischemia induce epigenetic modifications, in particular DNA demethylation, and their impact on RCC clinical epigenetic studies should be considered [50]. Another issue is the extent of tumor heterogeneity, which is expected to affect the results of epigenetic studies and the performance of potential biomarkers since subclones are likely responsible for progression and resistance to therapy [51]. From a methodological point of view, the vast majority of studies cannot be considered as evaluating acquired drug resistance along the course of systemic therapy but only inherent drug resistance since tissue samples originate from therapy-naive patients, and there are no publications reporting results from multiple tissue analyses at different time points along the patient clinical course [52]. In the current review, only one study [26] examined the dynamic changes in the VHL methylation status during therapy with sunitinib and concluded that these changes take place in the same way in both patient groups with favorable vs. poor therapy responses, which suggests no predictive value.

By definition, predictive biomarkers can help in the decision between two or more therapy options and/or can reveal the development of resistance along the course of the disease. Initially, biomarker discovery takes place through functional intervention studies, which include cell lines and animal models. This first step is necessary for investigating mechanisms of resistance to systemic agents and their dependence on epigenetic modifications. This technical validation should be followed by clinical validation to evaluate a potential predictive biomarker on the patient level. The best setting to achieve this clinical validation and further implementation of biomarkers in the treatment strategy is a randomized clinical trial (RCT) of targeted therapy vs. standard treatment or more treatments [53]. This multi-arm design is mandatory for sufficient demonstration of the predictive value of a potential biomarker because a biomarker that discriminates a patient group with a favorable clinical course from a cohort uniformly treated with the targeted therapy may be simply prognostic. Acquired resistance should be evaluated by longitudinal studies, which include comparing newly diagnosed tumors with matched samples taken at recurrence [54]. Up to now, there are only a few studies that examine acquired resistance by investigating longitudinal epigenetic changes pre- and post-therapy in various cancer entities. Considerations about obtaining multiple tumor samples along clinical courses or at the time of relapse present a clear barrier to longitudinal studies, but non-invasive methods of tumor sampling using circulating tumor cells or cell-free plasma DNA may help address this problem.

### 3.2. Limitations

Certain limitations were found in the present systematic review. At the study selection level, only articles in English were included. No defined criteria about the methodology, sample type, and size were applied in the selection process. The included studies were characterized by heterogeneity, and a number of them reported results relating to the association between gene methylation and drug sensitivity only as a secondary outcome. Another drawback is that the study design of several included publications does not exclude the possibility that methylation of a certain gene is just prognostic and not predictive.

At the outcome level, a major drawback in tissue studies is that many of the comparing groups are not adjusted for other factors affecting response to systemic therapy. Another issue is that outcome definition and reporting across studies are inconsistent. Only a number of studies report outcomes with measures of statistical significance. Conducting a quantitative synthesis of results was deemed inappropriate because outcomes are related to a variety of genes and are characterized by inconsistency.

## 4. Materials and Methods

### 4.1. Eligibility Criteria

This review is registered at the Open Science Framework (OSF) registry (registration DOI: https://doi.org/10.17605/OSF.IO/8PSQ4, accessed on 9 December 2023). In the selection process, articles with the following inclusion criteria were included: articles in English, all study designs, and only research articles on RCC with a focus on the correlation between methylation pattern of patient tissues and differences in clinical outcomes or RCC cell lines and cell sensitivity after delivery or exposure to systemic agents as chemotherapeutic drugs, immunotherapy, targeted substances. Studies reporting cell sensitivity data or clinical outcomes, which were retrieved only from bioinformatical databases, were excluded. No restrictions related to publication date, sample size, study design, or risk of bias were imposed.

Biologic material from patients, mainly normal and cancerous tissues, and various cell lines with known or unknown methylation patterns were used by the researchers to uncover possible correlations between modified methylation motifs and various degrees of response after exposure to systemic agents. The primary outcome measures included the correlation between hypermethylation of one or more genes and different therapy responses or different sensitivity for patients or RCC cell lines, respectively.

Using the PICOS approach, eligible studies on cell lines had the following characteristics: (i) population: RCC cell lines, (ii) intervention: treatment with a systemic agent with the gene under study in a methylated state, (iii) comparison: treatment with a systemic therapeutic agent with the gene under study in an unmethylated state, (iv) outcome: cell viability, apoptosis, cell cycle arrest, (v) study design: in vitro study. The respective characteristics for tissue studies were as follows: (i) population: patient RCC tissues, (ii) intervention: systemic therapy on patients with the gene under study in methylated state, (iii) comparison: systemic therapy on patients with the gene under study in unmethylated state, (iv) outcome: differences in clinical course, survival analysis, (v) study design: observational study

### 4.2. Literature Search Strategy

The majority of the studies were identified by searching four electronic databases (PubMed, Scopus, ScienceDirect, Web of Science). The search was performed by the authors of this article (A.K., M.P.) with an end-of-search date of 31 October 2023. No date range was set as a filter during the search. A few studies resulted from scanning the reference list of articles, especially reviews about biomarkers in RCC.

To maximize the discovery of articles related to epigenetic predictive biomarkers in RCC, we used a combination of search terms and Boolean operators. This combination was applied in the same manner in all 4 electronic databases and was synthesized as follows: “renal AND cancer” AND “DNA AND methylation” AND (biomarkers OR resistance OR sensitivity OR “epigenetic AND silencing” OR response).

### 4.3. Study Selection

Studies were selected and systematically reviewed following the PRISMA statement [55]. Eligibility assessment was conducted separately in an unblinded standardized manner by reviewers (A.K, M.P). The following terms were applied as exclusion criteria during the screening process at the abstract or full-text level: histone modification, noncoding RNA, prognostic biomarkers, diagnostic biomarkers, upper tract urothelial, cancer, and transitional cell cancer. Significant variation in the article evaluation was resolved by consensus. A full-text assessment was performed in the case of articles without a clear indication of exclusion during title and abstract appraisal, which took place on the records of the initial search.

### 4.4. Data Extraction Process

Reviewers created a data extraction sheet based on Microsoft Excel and initially tested on 8 randomly selected reports from the sum of included studies. After testing and refinement, A.K. performed data extraction, while M.P. checked the extracted data. Different aspects of some of the extracted data were resolved by discussion or reassessment by the third reviewer (G.D.).

Information was extracted from each included study on the (1) author and publication year, (2) gene under study, (3) molecular function of the gene, (4) systemic agent under study, (5) material under study, (6) study design, (7) testing on the control group, (8) results from electronic databases (TCGA), (9) molecular studies on cell lines (gene expression, protein expression), (10) functional studies on cell lines (DNMT inhibition, DNMT depletion, gene knockdown, gene vector-mediated enforced expression), (11) methylation status of the gene under study, (12) growth inhibition effect on cell cultures, (13) results of the application of RCC cells and systemic agents on xenografts, (14) results of cell sensitivity to systemic agents with respective evaluation methods, and (15) results of methylation-dependent patient clinical outcome with respective evaluation methods.

### 4.5. Risk of Bias in Individual Studies

The best method to evaluate a predictive biomarker for a targeted therapy is a prospective randomized clinical trial of the targeted therapy vs. a standard therapy, in which the biomarker status is evaluated for its predicting accuracy on both arms [56]. No such studies were discovered after a systematic search in electronic databases. Both reviewers considered the articles that included studies on patient tissues or cell lines, with a corroborating set of molecular tests that support the relationship between gene methylation status and response after exposure to a systemic agent.

Even though the evidence from these studies is not considered as strong as the respective results from randomized controlled trials, bias assessment is still necessary as a measure of outcome validity. Studies on tissues were appraised for bias by applying a scoring system based on the Newcastle–Ottawa Scale, which includes the same areas of interest (selection, comparability, and exposure or outcome) but slightly different criteria for each type of observational study (cohort, case-control, cross-sectional) [57]. Studies on cell lines were assessed by extending the application of the Office of Health Assessment and Translation (OHAT Risk of Bias Rating) Tool to mechanistic studies [58]. This bias assessment tool includes a four-grade rating for each of the nine criteria, which pertain to possible bias factors such as allocation to different exposures, experimental conditions, exposure characterization, outcome assessment, outcome report, and other threats to internal validity.

The risk of bias was appraised for each study independently by the two reviewers (A.K., M.P). In the case of a disagreement, the third reviewer (G.D) acted as a mediator. Bias assessment was performed only as a validity measure, and no studies were excluded because of a high risk of bias. Studies on both cell lines and tissues were assessed separately for the experimental part on cell lines and the observational part on tissues.

### 4.6. Outcome Measurements

Differences in cell sensitivity or therapy response in the context of different methylation patterns were the primary measure of the correlation between different biological behaviors to systemic agents and gene hypermethylation, suggesting the predictive value of the latest. The main summary effect measures in cell line studies included IC_50_ for cell growth inhibition, preG1 population for flow cytometry, and ratio of apoptotic cells for apoptosis assays. Respective measures in tissue studies included survival analysis and HR for patient cohorts with differences in methylation pattern, as well as methylation status in tissues of patients with different outcomes. Molecular tests were also considered to corroborate the relationship between gene methylation patterns and biological behavior.

### 4.7. Data Synthesis

Due to differences in genes and methodology among the included studies, statistical combination and meta-analysis of results were deemed inappropriate. Instead, outcomes were qualitatively appraised as demonstrating or not demonstrating a relationship between gene methylation and sensitivity/response to systemic agents.

## 5. Conclusions

The prediction of response to systemic therapy in RCC is an ongoing research problem that will continue to receive attention for many years. Despite the scarcity of studies on the role of methylation in predicting therapy response, there is evidence suggesting that such a relationship exists and that the development of a predictive clinical tool in the future is feasible. More mechanistic studies are required to shed light on the methylation of various genes in RCC and their correlation to drug effectiveness. Data from these experiments need to be validated in replication cohorts and prospectively assessed with longitudinal observations over time. Another intriguing possibility is predicting a therapy response by studying methylation in the blood, which suggests a non-interventional and more convenient method for personalizing treatment for the patient.

## Figures and Tables

**Table 1:** List of the included studies included in chronological order and their basic characteristics.

Author (Year)	Gene Name (as Stated in the Study)	Gene Name (As Stated in the Recent HUGO Nomenclature	Molecular Pathway/Function	Systemic Agent	Experimental Model	Study Design	Ref
To (2006)	*ABCG2*	*ABCG2*	ATP—binding cassette half transporter	Mitoxantrone, topotecan, SN38	cell lines	experimental (cell lines)	[13]
Reu (2006)	*RASSF1*	*RASSF1*	Death receptor-dependent apoptosis	Interferons	cell lines	experimental (cell lines)	[14]
Reu (2006)	*XAF1*	*XAF1*	Interferon-induced apoptosis	Interferons	cell lines	experimental (cell lines)	[15]
Lee (2006)	*XAF1*	*XAF1*	Binding to and counteracting the inhibitory effect of XIAP	Etoposide, 5-FU	cell lines, patient tissues	experimental (cell lines), cross-sectional (tissues)	[16]
Shen (2007)	32 promoter CpG islands		Respective pathways	170 agents	cell lines	experimental (cell lines)	[17]
Takano (2010)	*Cx32*	*GJB1*	GJ-dependent transfer of small molecules	Vinblastine	cell lines	experimental (cell lines)	[18]
Dubrowinskaja (2013)	*NEFH*	*NEFH*	Type IV intermediate filament protein	Anti—VEGF agents	Cell lines, patient tissues	experimental (cell lines), cohort (tissues)	[19]
Choueiri (2013)	*VHL*	*VHL*	VHL-HIF pathway	Pazopanib	patient tissues	cohort (tissues)	[20]
Weygant (2014)	*DCLK1*	*DCLK1*	EMT, stemness regulation	Sunitinib	cell lines, patient tissues, databases	experimental (cell lines), cross-sectional (tissues)	[21]
Peters (2014)	*CST6, LAD1*	*CST6, LAD1*	CST6 (cysteine protease inhibitor), LAD (stability of the epithelial–mesenchymal interaction)	sunitinib, sorafenib, axitinib, bevacizumab	patient tissues	cohort (tissues)	[22]
Motzer (2014)	*VHL*	*VHL*	VHL-HIF pathway	Sunitinib	patient tissues	cohort (tissues)	[23]
Ponnusamy (2015)	*MSH2*	*MSH2*	MMR-dependent apoptosis	doxorubicin, cisplatin	cell lines	experimental (cell lines)	[24]
Kim (2015)	*FLT1, KDR*	*FLT1, KDR*	VEGF-VEGFR signaling	Bevacizumab, sunitinib, axitinib, anti-FLT1 peptide, anti-KDR antibody	cell lines, patient tissues	experimental (cell lines), cohort (tissues)	[25]
Stewart (2015)	*VHL*	*VHL*	VHL-HIF pathway	Sunitinib	patient tissues	case-control (tissues)	[26]
Liu (2015)	*ASC/TMS1*	*PYCARD*	Caspase-9 dependent apoptosis	doxorubicin, etoposide	cell lines, patient tissues	experimental (cell lines), cross-sectional (tissues)	[27]
Nogales (2015)	*SLFN11*	*SLFN11*	Inhibition of DNA replication in response to DNA damage	Cisplatin, carboplatin	cell lines	experimental (cell lines)	[28]
Beuselinck (2015)	Multiple genes		Respective pathways	Sunitinib	patient tissues	cohort (tissues)	[29]
Liu (2016)	*OCT2*	*SLC22A2*	Polyspecific organic cation transporter	Oxaliplatin	cell lines, patient tissues	experimental (cell lines), cross-sectional (tissues)	[30]
Zhou (2016)	*DAB2IP*	*DAB2IP*	Ras -GTPase activation	mTOR inhibitors	cell lines, patient tissues	experimental (cell lines), cohort(tissues)	[31]
Winter (2016)	Multiple pharmacogenes		Respective pathways	Cisplatin	cell lines, patient tissues	experimental (cell lines), cross-sectional (tissues)	[32]
Pompas-Veganzones (2016)	*SYNPO2*	*SYNPO2*	Actin-binding and actin -bunding activity	Antiangiogenic agents	patient tissues	cohort (tissues)	[33]
Wang (2017)	*ASPP1*	*ASPP1*	Apoptotic stimulation of P53 protein	5-FU	cell lines, patient tissues	experimental (cell lines), cross-sectional (tissues)	[34]
Verbiest (2018)	Multiple genes		Respective pathways	Pazopanib	patients tissues	cohort (tissues)	[35]
Lei (2018)	*LIFR*	*LIFR*	Signal transduction of the IL-6	Verteporfin, PHA—665752, PF—2341066	cell lines, patient tissues	experimental (cell lines), cross-sectional (tissues)	[36]
Kammerer (2018)	*VHL*	*VHL*	VHL-HIF pathway	Sunitinib	patient tissues	case-control (tissues)	[37]
Li (2019)	*PON1*	*PON1*	Ca^2+^-dependent high-density lipoprotein”	Sunitinib	cell lines, patient tissues	experimental (cell lines), cross-sectional (tissues)	[38]
Zhao (2019)	*QPCT*	*QPCT*	Posttranslational protein modification	Sunitinib	cell lines, patient tissues	experimental (cell lines), cohort (tissues)	[39]
De Cubas (2020)	Transposable elements (TE)		Endogenous retroviruses activating antiviral signaling	PD-1/PD-L1	cell lines, patient tissues	experimental (cell lines), cohort (tissues)	[40]
Miyakuni (2021)	*UQCRH*	*UQCRH*	Mitochondrial complex III component	Everolimus	cell lines, patient tissues	experimental (cell lines), cross-sectional (tissues)	[41]
Klümper (2021)	*CTLA4*	*CTLA4*	Immune checkpoint receptor	Immune checkpoint inhibitors	patient tissues	cohort (tissues)	[42]
Ye (2022)	*TCAIM*	*TCAIM*	Priming capacity and activation of T cells	Sunitinib	cell lines, patient tissues	experimental (cell lines), cross-sectional (tissues)	[43]

Abbreviations: HUGO: Human Genome Organisation; GJB1: Gap junction protein beta 1; PYCARD: PYD And CARD Domain Containing; SLC22A2: Solute Carrier Family 22 Member 2; ATP: Adenosine triphosphate; XIAP: X-linked inhibitor of apoptosis protein; 5-FU: 5-Fluoruracil; VEGF: Vascular endothelial growth factor; VHL-HIF: Von Hippel Lindau-Hypoxia Inducible Factor; EMT: Epithelial–mesenchymal transition; MMR: DNA mismatch repair; IL-6: Inteleukin-6; PD-1/PD-L1: Programmed cell Death1/Programmed cell Death-Ligand1.

## Data Availability

Not applicable.

## Supplementary Materials

The following supporting information can be downloaded at https://www.mdpi.com/article/10.3390/epigenomes8030028/s1, Table S1: Distribution of included articles in electronic databases. Table S2: Summary of the results from experiments performed with renal cancer cell lines. Table S3: Summary of the results from tissue studies. Table S4: OHAT rating tool for assessment of risk of bias in mechanistic studies. Table S5: Newcastle–Ottawa scale for assessing risk of bias in observational studies. Table S6: Origin of the cell lines used for in vitro analyses in the included studies of the review. Table S7: Histological categorization of the analyzed tissue samples of the included studies. Figure S1: PICOS framework on RCC cell line studies. Figure S2: PICOS framework on tissue studies.
