# Supplementary material for: DNA Methylation as Drug Sensitivity Marker in RCC: A Systematic Review
Source: Epigenomes. 2024 Jul 15;8(3):28. doi: 10.3390/epigenomes8030028 (PMC11270435; doi:10.3390/epigenomes8030028)
Supplement: Supplementary file 1 [file epigenomes-08-00028-s001.zip › epigenomes-2960687-supplementary.pdf]

Table S1. Distribution of included articles in electronic databases.

Author (year)	Reference	Gene	pubmed	scopus	science direct	web of science	Reference lists
To (2006)	[13]	*ABCG2*	✓	✓		✓	
Reu (2006)	[14]	*RASSF1*			✓	✓	
Reu (2006)	[15]	*XAF1*			✓	✓	
Lee (2006)	[16]	*XAF1*			✓		
Shen (2007)	[17]	32 promoter CpG islands	✓	✓		✓	
Takano (2010)	[18]	*Cx32*			✓		
Dubrowinskaja (2013)	[19]	*NEFH*	✓	✓		✓	
Choueiri (2013)	[20]	*VHL*	✓	✓		✓	
Weygant (2014)	[21]	*DCLK1*			✓		
Peters (2014)	[22]	*CST6, LAD1*		✓			
Motzer (2014)	[23]	*VHL*					✓
Ponnusamy (2015)	[24]	*MSH2*		✓			
Nogales (2015)	[28]	*SLFN11*					✓
Kim (2015)	[25]	*FLT1, KDR*		✓			
Liu (2015)	[27]	*ASC/TMS1*	✓	✓		✓	
Stewart (2015)	[26]	*VHL*					✓
Beuselinck (2015)	[29]	Multiple genes		✓			
Liu (2016)	[30]	*OCT2*	✓			✓	
Zhou(2016)	[31]	*DAB2IP*					✓
Winter (2016)	[32]	Multiple pharmacogenes					✓
Pompas - Veganzones (2016)	[33]	*SYNPO2*	✓	✓			
Wang (2017)	[34]	*ASPP1*	✓	✓			
Verbiest (2018)	[35]	Multiple genes	✓	✓	✓	✓	
Lei (2018)	[36]	*LIFR*			✓	✓	
Kammerer (2018)	[37]	*VHL*			✓		
Li (2019)	[38]	*PON1*	✓				
Zhao (2019)	[39]	*QPCT*	✓				
De Cubas (2020)	[40]	Transposable elements (TE)	✓			✓	
Miyakuni (2021)	[41]	*UQCRH*	✓	✓	✓		
Klümper (2021)	[42]	*CTLA4*	✓	✓	✓	✓	
Ye (2022)	[43]	*TCAIM*		✓	✓		

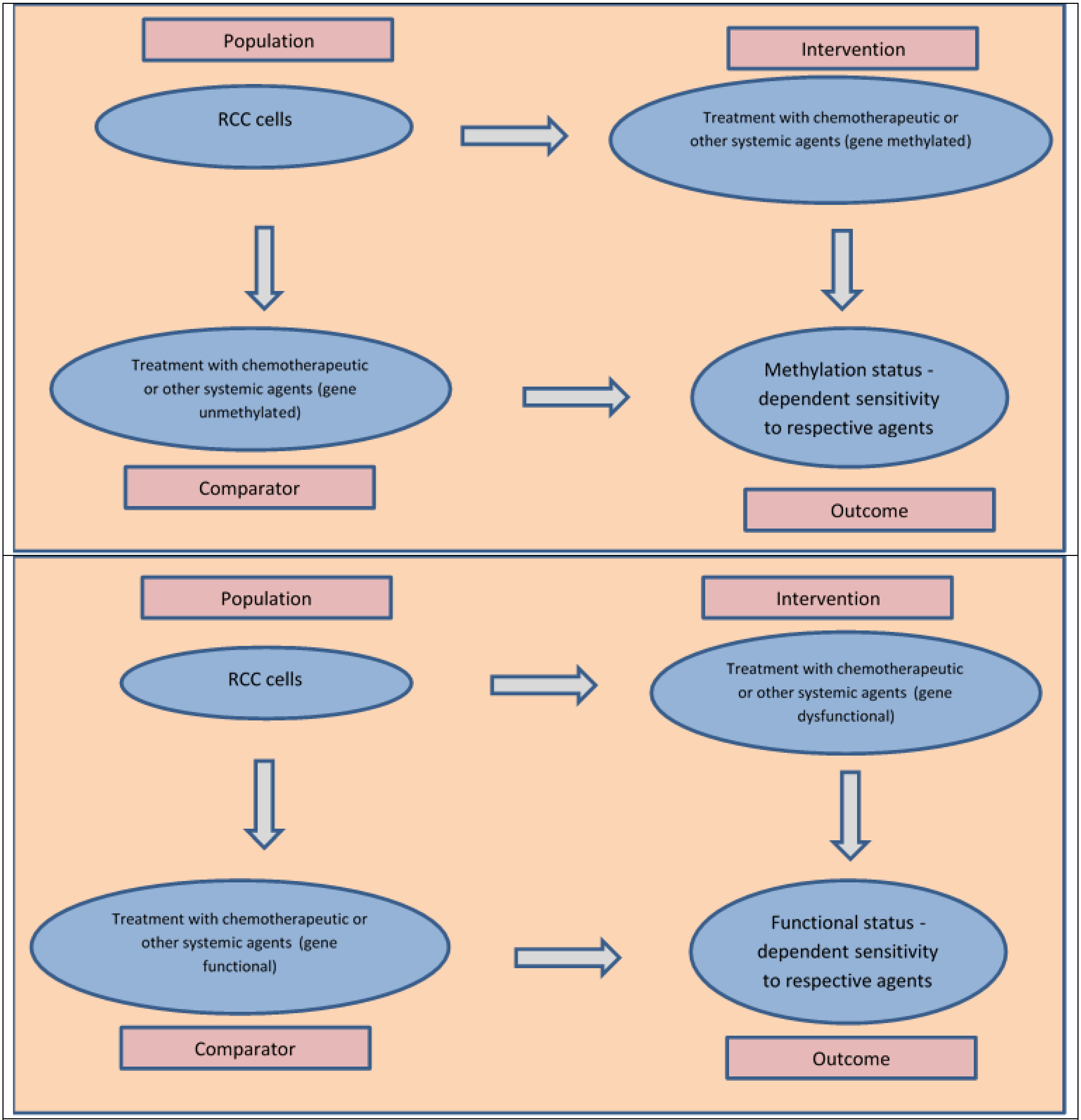

Figure S1. PICOS framework on RCC cell line studies.

Cohort design

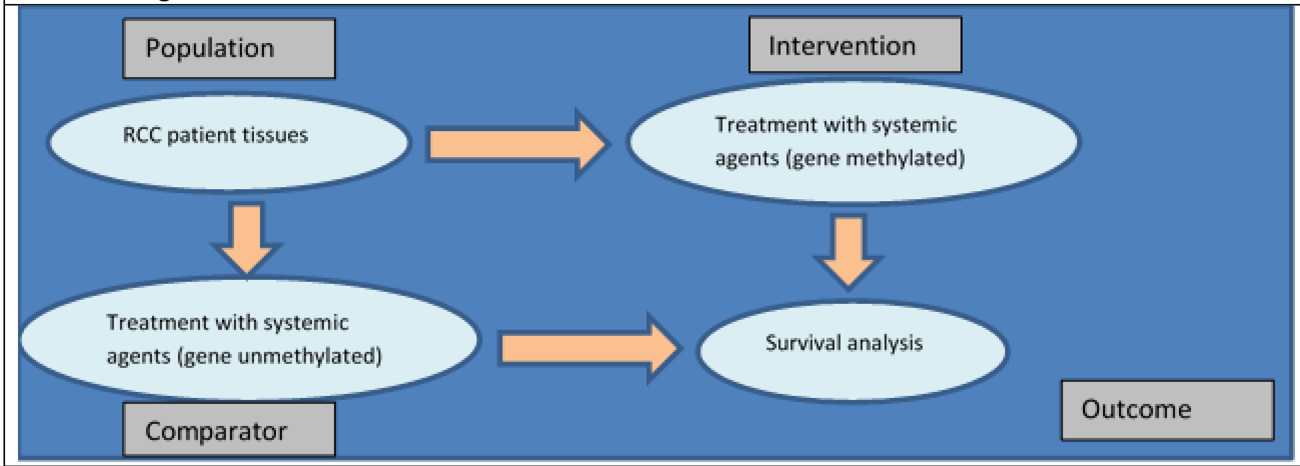

Case control design

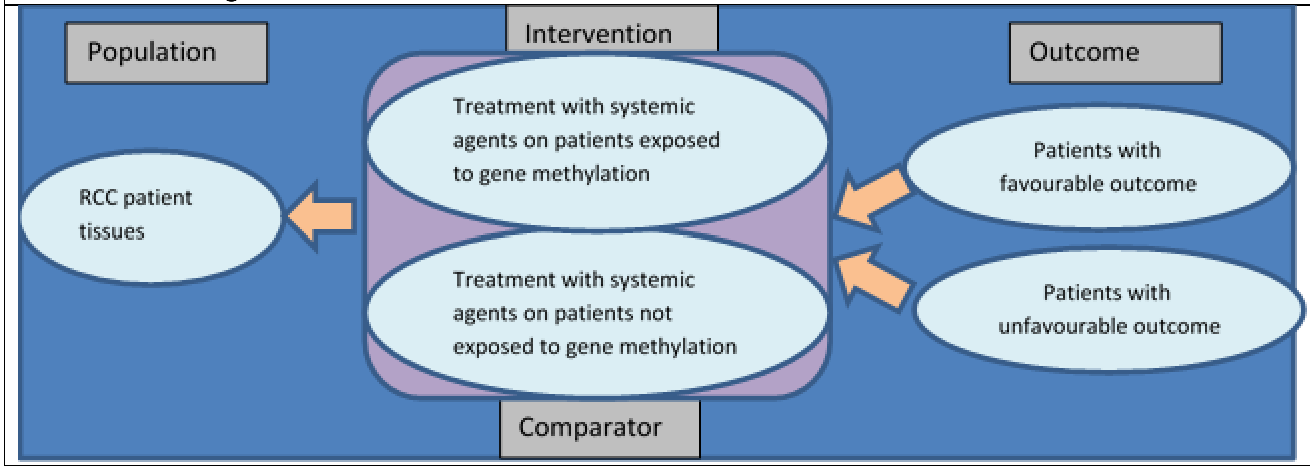

Cross-sectional design

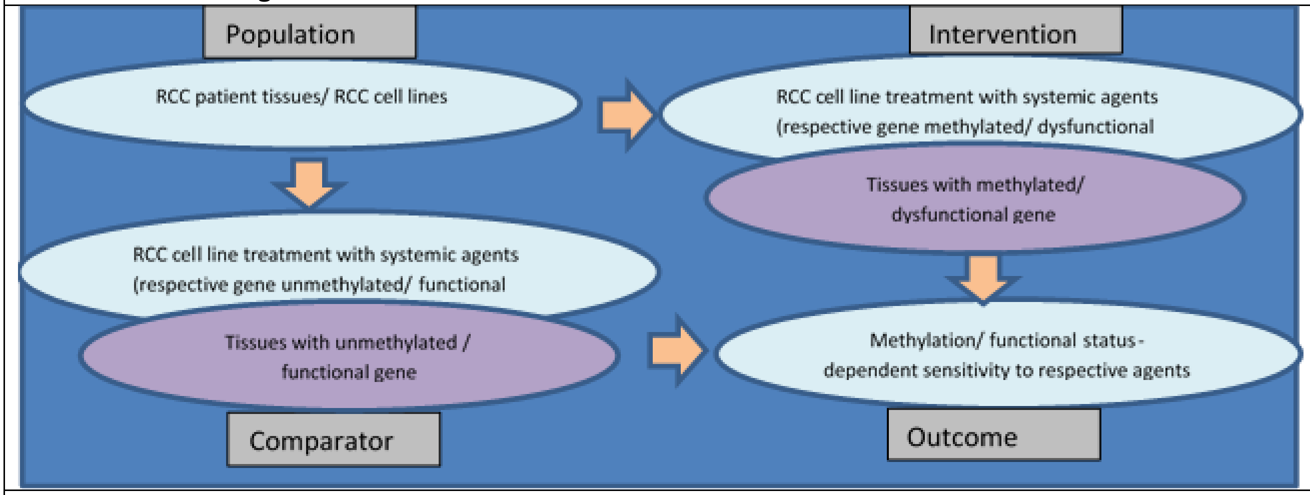

Figure S2. PICOS framework on tissue studies.

Table S2. Summary of the results from experiments performed with renal cancer cell lines.

Gene (ref)	Experimental model	Molecular studies/ *Functional studies*	Methylation status	Evaluation method for drug sensitivity	Methylation status - dependent sensitivity to respective drugs	Functional status - dependent sensitivity to respective drugs
ABCG2 [13]	3 RCC lines (UOK121, UOK143, UOK181)	Gene expression<br>Methylation status /*DNMT inhibition*	UOK121, UOK143: methylated, UOK181: unmethylated	Cytotoxicity assay (IC_{50})	Mitoxantrone IC_{50} (UOK121= 36.4 +/- 4, UOK143= 47.1 +/- 10.3, UOK181= 304.2 +/- 84.4), Topotecan IC_{50} (UOK121= 39.0 +/- 3.6, UOK143= 52.9 +/- 7.5, UOK181= 1075.0 +/- 196.1), SN-38 IC_{50} (UOK121= 47.9 +/- 0.8, UOK143= 38.5 +/- 5.6, UOK181= 3918.0 +/- 373.1)	*ABCG2* inhibition with FTC reduced IC_{50} of UOK181 by 66% under treatment with any of 3 chemotherapeutics. No impact on IC_{50} of UOK121, and UOK143 under the same chemotherapeutics.
RASSF1 [14]	2 RCC lines (ACHN, SK-RC-45), 1 control line (normal kidney epithelial cells)	Gene expression<br>Protein expression<br>Methylation status (tissues)<br>*/DNMT inhibition*<br>*DNMT depletion*<br>*Gene knockdown*<br>*Gene enforced expression*		Apoptosis assay (TUNEL)	ACHN and SK-RC-45 cells were resistant under high-dose IFN treatment, while pretreatment with DNMT1 inhibitor increased apoptotic response to IFN by up to 70%. Apoptotic fraction of SK-RC-45 increased from 5.4 +/- 4.5% to 23.4+/-12% (IFN-a2) and 45+/- 1% (IFN-β) after DNMT1 depletion.	*RASSF1* inhibition by siRNA on DNMT1 AS pretreated ACHN cells decreased IFN-induced apoptosis from 63.9+/-9.19% to 35+/-4.1%. Enforced *RASSF1* expression by lentivirus infection increased IFN-induced apoptosis in ACHN cells from 3.77 +/- 1.67% to 16.83 +/- 0.98%.
XAF1 [15]	3 RCC lines (ACHN, SK-RC-45, SK-RC-29), 2 control lines (NKE39, NKE58)	Gene expression<br>Protein expression<br>Methylation status<br>*/DNMT inhibition*<br>*DNMT depletion*<br>*Gene knockdown*<br>*Gene enforced expression*	ACHN: methylated, SK-RC45: unmethylated	Growth inhibition assay (SRB), Apoptosis assay (TUNEL)	DNMT inhibition (decitabine) or DNMT depletion (AS) increased IFN-induced apoptosis up to 83 % of RCC cells.	*XAF1* knocking down combined with decitabine reduced IFN-induced apoptosis from 59 % (for cells without *XAF1* siRNA) to 18%. Enforced *XAF1* expression resulted in more than 80% apoptotic ACHN under a high IFN-β dose (500 U/ml).
p73[17]	7 RCC lines (UO-31, SN12C, A498, Caki-1, 786-O, ACHN, TK-10)	Gene expression<br>Protein expression<br>Methylation status<br>*/Gene knockdown*	Values per gene and cell line (table), TK10: expressing p73, unmethylated for p73,786-O : weakly expressing p73, methylated for p73	Sensitivity pattern per cell line (IC_{50}) plus correlative analysis of methylation per gene against systemic agents (Pearson coefficient, two-tailed p), Flow cytometry before/ after cisplatin treatment.	Respective tables, higher sensitivity (GI_{50} = 1 μmol/L) of 786-O cells (methylated) compared to TK10 (GI_{50} = 12.5 μmol/L) (unmethylated).	*p73* knocking down induced higher sensitivity to cisplatin in both cell lines (greater increase in unmethylated TK10, GI_{50} decreased from 12.5 to 2.5 μmol/L).
Cx32 [18]	1 RCC line (Caki-1)	Protein expression<br>*/DNMT inhibition*<br>*Gene knockdown*		Proliferation assay, flow cytometry, caspase 3 activity	Cell viability under vinblastine > 10nM was reduced after pretreatment with decitabine, which reactivated *Cx32* expression, SubG1 population maximally increased after vinblastine treatment on pretreated with decitabine cell population.	Vinblastine-induced cytotoxicity significantly decreased after *Cx32* knockdown by siRNA (60% reduction in the increased by decitabine caspase 3 activity).
MSH2 [24]	3 RCC lines (Caki -1, Caki-1^{LA}, Caki-1^{HA})	Gene expression<br>Protein expression<br>Methylation status<br>*/DNMT inhibition*	Methylation increased from 5% in Caki-1 to 9% in Caki-1^{LA} (chemoresistant cell line).	MTT assay, flow cytometry	Cytotoxicity (doxorubicin) was higher in Caki-1 (35%) than in Caki-1^{LA} (16%) or Caki-1^{HA} (25%). Same effect after cisplatin (51% in Caki-1, 17% in Caki-1^{LA}). PreG1 cells after doxorubicin were 33% for Caki-1, 11% for Caki-1^{LA}, and 22% for Caki-1^{HA}. Cytotoxity increased after treatment with decitabine plus doxorubicin: from 35 to 48% in Caki-1, from 15 to 26% in Caki-1^{LA}, and from 25 to 41 % in Caki-1^{HA}. Increase in apoptotic preG1 population: from 11 to 34 % in Caki-1^{LA} and from 22 to 24% in Caki-1^{HA}.	

SLFN11 [28]	8 RCC lines (TK10, A498, UO31, SN12C, 786-O, Caki1, RFX393, ACHN)	Methylation status	Average methylation (beta value) High methylation: TK10 (0.88), A498(0.73) Low methylation: UO31(0.28), SN12C(0.08), 786-O (0.07), Caki1(0.07), RFX393(0.06), ACHN(0.04).	Cell viability assay (IC_{50} value= -log[M])	1st value= IC_{50} carboplatin, 2nd value= IC_{50}cisplatin 786-O(4.0, 6.0), A498(3.7, 5.1), ACHN (4.1, 5.9), Caki-1 (4.0, 5.7), RFX393 (4.1, 5.5), SN12C (3.8, 5.3), TK10 (3.7, 4.9), UO31 (3.8, 5.5).	
XAF1 [16]	15 RCC lines (UOK101, UOK105, UOK107, UOK108, UOK109, UOK110, UOK112, UOK115, UOK121, UOK122, UOK123, UOK124, UOK130, ACHN, WWC1)	Gene expression Methylation status Mutation status / *DNMT inhibition* *Gene knockdown* *Gene enforced expression*	10 out of 15 cell lines had no or very low *XAF1* expression and proved to be methylated, 5 cell lines with normal expression had no methylation.	Apoptosis assay (TUNEL)		253-J non-*XAF1* expressing cells showed a significant apoptosis response after enforced *XAF1* expression plus 5-FU or etoposide, compared to chemotherapeutics alone (p<0.01). HT1376 expressing *XAF1* cells showed decreased apoptotic response after *XAF1* knocking down plus chemotherapeutics, compared to no knocking down plus chemotherapeutics (p<0.05).
NEFH [19]	6 RCC line (RCC-MF, RCC-HS, RCC-GS, ACHN, A498, 786-O), 1 control line,	Methylation status *expression*	>25% relative methylation in 2 of 6 RCC lines.			
FLT1, KDR [25]	13 RCC lines (A498, A704, ACHN, Caki-1, Caki-2, SN12C, SNU1272, SNU228, SNU333, SNU349, SNU482, SNU267) , 3 control lines (HUVEC, SNU1, H460),	Gene expression Methylation status /*DNMT inhibition* *Gene knockdown*	low *FLT1/ KDR* meth: SNU482, SNU 228, high *FLT1*/low *KDR* meth: SN12C, SN12PM6, low *FLT1/* high *KDR* meth: A704, ACHN, Caki-1, SNU1272, high *FLT1/KDR*: SNU333, SNU349, A498, SNU267.	Proliferation assay (CCK8 assay)	Cell lines with high *FLT1* methylation showed lower proliferation-inhibitory effects under treatment with anti-*FLT1* peptide (t-value=5.28, p<0.0001), sunitinib (t-value=4.77, p<0.0001) and axitinib (t-value=3.78, p=0.0005) compared to RCC cells with low *FLT1* methylation.	Decrease of growth inhibition in *FLT1* -knockdown SNU482 by anti-*FLT1* peptide (t-value=6.04, p<0.0001), sunitinib (t-value= 6.87, p<0.0001), axitinib (t-value= 5.09, p<0.0001), no change for bevacizumab, anti-*KDR* antibody.
ASC/TMS1 [27]	6 RCC lines (A498,786-O, Caki-1, Caki-2, Kert-3, 769P), 1 control line (HEK293),	Gene expression Protein expression Methylation status /*DNMT inhibition* *Gene knockdown* *Gene enforced expression*	All 6 RCC cell lines *ASC/TMS1* silenced or downregulated proved to be fully or partially methylated.	Cell viability assay (CCK-8 assay)	Diminished 786-O cell viability after demethylating pretreatment and treatment with either etoposide (p<0.05) or doxorubicin (p<0.05) compared to without demethylating pretreatment.	Diminished 786-O cell viability after enforced *ASC/TMS1* expression and treatment with either etoposide (p<0.05) or doxorubicin (p<0.05) compared to without enforced *ASC/TMS1* expression pretreatment. Caki-2 cells after *ASC/TMS1* knocking down and treatment with etoposide or doxorubicin, showed diminished *p53* activation, which suggests reduced apoptosis.

OCT2 [30]	3 RCC lines (769-P, 786-O, Caki-1),	Gene expression<br>Protein expression<br>Methylation status<br>*/DNMT inhibition*<br>*Gene knockdown*	All RCC cell lines were hypermethylated.	Proliferation assay (IC_{50}), Relative tumor volume in xenografts (curve)	13-, 6-, 4-fold reduction of oxaliplatin IC_{50} after pretreatment with decitabine in 769-P, 786-O, Caki-1 cell line respectively. Combined treatment with decitabine and oxaliplatin delayed tumor growth and resulted in >50% tumor shrinkage in the Caki-1 xenograft model.	No reduction of oxaliplatin IC_{50} after pretreatment with decitabine for cell lines with *OCT2* shRNA (*OCT2* knockdown).
DAB2IP [31]	6 RCC lines (769-P, 786-O, A498, ACHN, Sut002, Sor001) , 2 control lines (HK-2, MDCK)	Gene expression<br>Protein expression<br>Methylation status (tissues)<br>*/DNMT inhibition*<br>*Gene knockdown*		MTT assay (IC_{50}), tumor enlargement in xenografts (mm3)		After temsirolimus treatment, increased sensitivity of 786-O Con ($p<0.05$) and Sut002 DAB2IP ($p<0.05$) compared to 786-OKD and Sut002VC respectively. Profile of *DAB2IP* mRNA levels and IC_{50} across RCC cell lines revealed an inversed correlation. Faster tumor enlargement under temsirolimus treatment in xenografts with 786-OKD ($p<0.05$) and Sut002VC ($p<0.05$).
Multiple pharmaco genes , *OCT2* [32]	5 RCC lines (A-498, 786-O, Caki-1, Caki-2, ACHN)	Gene expression<br>Protein expression<br>Methylation status<br>*/DNMT inhibition*	All RCC cell lines were hypermethylated in the *2 OCT2* promoter region.	Flow cytometry (% survival)	Significantly increased induction of apoptosis to Caki-2 cells treated with decitabine plus cisplatin compared to cisplatin alone ($p<0.01$, ANOVA with Newman-Keuls Post Test for multiple comparisons).	
ASPP1 [34]	4 RCC lines (786-O, 769-P, Caki-1, CCF-RC1) 1 control cell line (HEK293)	Gene expression<br>Protein expression<br>Methylation status<br>*/DNMT inhibition*<br>*Gene knockdown*<br>*Gene enforced expression*	Methylation rate for HEK292, 769-P, 786-O, CCF-RC1, Caki-1 was 11%, 8%, 63%, 42%, 39% respectively.	Proliferation assay (MTT), apoptosis assay		786-O/ vector showed a higher IC_{50} value (twofold, $p <0.05$) compared to 786-O/*ASPP1*. *ASPP1* knockdown doubled IC_{50} value in 786-P ($p <0.01$) and Caki-1 ($p <0.05$) cells. Apoptosis reduced after *ASPP1* knockdown in 786-P ($p <0.05$) and Caki-1 ($p <0.05$) cells.
LIFR [36]	2 RCC lines (786-O, Caki-2)	Gene expression<br>Protein expression<br>Methylation status (tissues)<br>*/DNMT inhibition*<br>*Gene knockdown*		MTT assay (IC_{50}), Spearman correlation (between mRNA levels and sensitivity data from 24 anticancer drugs on 9 RCC cell lines, CCLE)		Knocking down the *LIFR* gene in Caki-2 cells resulted in a sensitivity increase (IC_{50} value from 16.91 to 10.38 μmol), Strong correlation between *LIFR* mRNA level and drug sensitivity for anticancer agents PHA-665752 ($r= 0.707$, $p=0.033$), PF2341066 ($r=0.707$, $p=0.033$).
DCLK1 [21]	1RCC line (Caki-2),	Gene expression<br>Protein expression<br>*/Gene knockdown*		Proliferation assay		30% viability reduction after gene knocking down ($p<0.002$).
PON1 [38]	3 RCC lines (786-O, Caki-2, SKRC39), 1 control cell line (HK-2)	Gene expression<br>Protein expression<br>Methylation status	Hypermethylation in 786-O ($p<0.01$), Caki-2 ($p<0.01$), SKRC39 ($p<0.001$) compared to HK-2 cells	Proliferation Assay (MTT), flow cytometry, Apoptosis assay (TUNEL)<br>tumor enlargement in xenografts (mm3)	Stronger inhibition of cell proliferation under sunitinib + decitabine ($p<0.05$), more cells arrested at G0/G1 phase under sunitinib + decitabine ($p<0.01$), apoptotic cells increased under sunitinib + decitabine compared to treatment with sunitinib. Inhibition of tumor growth in xenografts under sunitinib + decitabine ($p<0.05$) compared to sunitinib monotherapy (paired t-test).	

QPCT [39]	8 RCC cell lines (OS-RC-2, Caki-2, Caki-1, A498, 798-O, ACHN, 769-P, KETR-3), 1 control cell line (HK-2)	Gene expression, protein expression, methylation status/ *DNMT inhibition, gene knockdown, gene enforced expression.*	Reduced methylation across various gene regions with increased gene expression after treatment of RCC cells with decitabine.	Proliferation assay (CCK8 assay), cytotoxicity assay (IC_{50}), colony formation assay, flow cytometry		RCC cell lines with *QPCT* suppression had a reduced IC_{50} ($p<0.01$) and more apoptotic cells ($p<0.01$). Enforced gene expression resulted in a higher IC_{50} ($p<0.01$) and the formation of more colonies ($p<0.01$).
Transposable elements (TE) [40]	3 RCC cell lines (786-O, A498, UMRC2), 2 control cell lines (RPTeC, HKC).	Gene expression, Methylation status/ *DNMT inhibition*	After DNMT inhibition TE hypomethylation with increased expression			
UQCRH [41]	4 RCC cell lines (OS-RC-2, OS5K-1, OS5K-2, OS5K-3), 2 control cell lines (HK-2, 293FT).	Gene expression, protein expression, methylation status/ *DNMT inhibition, gene knockdown, gene enforced expression.*	Highly malignant OS5K cell group was methylated, OS-RC-2 unmethylated.	Apoptotic cell detection by flow cytometry	More intense everolimus-induced apoptosis in OS-RC-2 than in OS5K cells. Pretreatment with decitabine enhanced apoptosis in OS5K cells.	
TCAIM [43]	6 RCC cell lines (786-O, OS-RC-2, Caki-1, A498, 769-P, ACHN), 1 control cell line (HK-2)	Gene expression, protein expression/ *gene knockdown, gene enforced expression.*		Cell viability assay (CCK-8 assay), Apoptotic cell detection by flow cytometry		Gene-enforced expression increased sensitivity to sunitinib compared to control ($p<0.0001$). Gene silencing decreased sensitivity to sunitinib compared to control ($p<0.0245$).

Abbreviations: RCC: Renal cell cancer; DNMT: DNA methyltransferase; IC_{50}: Half-maximal inhibitory concentration; FTC: Fumitremorgin C; siRNA: Small interfering RNA; TUNEL: TdT-mediated dUTP-biotin nick end labeling; IFN: Interferone; SRB: Sulforhodamine B; MTT: 3-(4,5-dimethylthiazolyl-2)-2,5 diphenyltetrazolium bromide; CCK-8: Cell Counting Kit-8; ANOVA: Analysis of variance; shRNA: short hairpin RNA

Table S3. Summary of the results from tissue studies

Gene (ref)	Materials	Systemic agent	Methylation status	Evaluation method for methylation-dependent therapy response	Outcome
XAF1 [16]	20 tumor tissues, 20 normal tissues (fresh frozen tissues)	Etoposide, 5-FU	6 out of 7 RCC tissues with low *XAF1* expression showed methylation, all normal and tumor tissues with normal *XAF1* expression showed no methylation.		
NEFH [19]	114 tumor tissues, 83 normal tissues (fresh frozen tissues), 18 tumor tissues (FFPE)	Anti - VEGF agents	7.3 fold increase in relative methylation of cancerous tissues (0.634% vs. 0.087% in normal tissues, p<0.001) (paired t-test).	Survival analysis (Kaplan -Meier plot), log-rank statistics.	Median OS of 29.8 vs. 9.8 months for patients with low and high methylation respectively (p=0.028). By using a cutoff of 6 months for PFS, *NEFH* methylation detects therapy failure with a sensitivity of 0.91 (0.62-0.98, 95%CI).
FLT1, KDR [25]	8 tumor tissues, 8 normal tissues (fresh frozen tissues), 13 tumor tissues (FFPE)	Bevacizumab, sunitinib, axitinib, anti *FLT1* peptide, anti *KDR* antibody	Methylation level in normal (n) and tumor (t) tissue:<br>*FLT1*: 1.3% (n), 4.4% (t), p= 0.023 *KDR*: 2.2% (n), 16.4% (t), p=0.008 (Wilcoxon's singed-rank test).	Methylation status in responders vs. non-responders (paired t-test)	*FLT1* methylation significant higher (p= 0.030) in non-responders (19.48 +/- 13.15 %) than in responders (6.84 +/- 1.19 %).
ASC/ TMS1 [27]	202 tumor tissues, 25 normal tissues (fresh frozen tissues)	Doxorubicin, etoposide	*ASC/TMS1* proved to be tumor-specific methylated in RCC, with 83/202 (41.1%) of tumor samples and 3/25 (12%) of normal tissues methylated.		
OCT2 [30]	46 tumor tissues, 46 normal tissues (fresh frozen tissues), 31 tumor tissues, 31 normal tissues (tissue microarray)	Oxaliplatin	Significant methylation percentage increase for tumor tissues with strong *OCT2* repression (p<0.001) and weak repression (p=0.006). (two-tailed paired t-test).		
DAB2IP [31]	439 tumor tissues, 40 normal tissues (tissue microarray), TCGA data	mTOR inhibitors	(only protein expression was studied in tissues of the patient cohort, *DAB2IP* expression was detected in 38/40 normal kidney tissues, and decreased in 239/439 RCC tissues), 82% of the tumor tissues methylated according to TCGA data.	Kaplan Meier analysis (log-rank statistics)	(Median survival of 46 vs. 23.6 months for patient subset (n=17) after mTOR treatment with high vs low *DAB2IP* expression, HR=1.67, p=0.41, TCGA data).
Multiple pharmacog enes, *OCT2* [32]	34 (primary) + 20 (metastatic) tumor tissues (fresh frozen tissues)	Cisplatin	Comparing primary tumors with metastases, only a few significantly differentially methylated CpG sites were identified after adjustment for multiple testing, 41,682 and 8,194 differently methylated CpG sites (adjusted p < 1E-05) were found after comparison of cell lines to primary ccRCC or metastases respectively. No significant differences in the *OCT2* promoter methylation between metastasis and tumor tissue.		
ASPP1 [34]	20 matched tumor-normal tissues (fresh frozen tissues) 94 tumor tissues, 13 normal tissues (tissue microarray)	5-FU	(Only mRNA and protein expression were studied on tissues).		
LIFR [36]	25 tumor tissues, 25 normal tissues (FFPE), TCGA data	Verteporfin, PHA-665752, PF 2341066	(Only mRNA and protein expression were studied on tissues).<br>TCGA (Higher methylation levels in 3 CpG sites in tumor vs. normal samples (p<0.001), negative correlation between DNA methylation and gene transcription.)		
DCLK1 [21]	172 tumor tissues, 20	Sunitinib	(only immunohistochemistry was performed on tissues),		

	normal tissues, (tissue microarray) TCGA data.		TCGA (AUC= 0.838 +/- 0.024 for β- promoter, ROC curve analysis).		
VHL [20]	78 tumor tissues (FFPE)	Pazopanib	8 of 78 samples showed hypermethylation.	Chi-square exact test, Kaplan Meier analysis	*VHL* gene status did not correlate with either ORR (37.5% vs. 41.4%, p= 0.1673), or median PFS (13.8 vs. 17.4 months) in patients with vs. without *VHL* inactivation).
CST6, LAD1 [22]	18 tumor tissues (FFPE)	Sunitinib, sofanenib, axitinib, bevacizumab	8/18 tissues relatively hypermethylated for *LAD1*, 10/18 tissues relatively hypermethylated for *CST6*.	PFS - OS (Kaplan Meier plot), PFS–OS differences (log-rank statistics), HRs (Univariate Cox regression), Sensitivity - specificity (PFS cutoff=6 months)	Shortened PFS for hypermethylated *CST6* and *LAD1* (log-rank p=0.009 and p=0.004, HR= 4.1(1.2- 12.6, 95%CI) and 6.4 (1.6- 26.0, 95%CI), Shortened OS for hypermethylated *CST6* and *LAD1* (log-rank p=0.011 and p=0.043, HR= 4.1 (1.3- 13.4, 95%CI) and 2.9 (1.0- 8.6, 95%CI), prediction accuracy for therapy failure: AUC *CST6*= 0.88, AUC *LAD1*= 0.90.
VHL [23]	143 tumor tissues (FFPE)	Sunitinib	14 RCCs methylated/ 109 RCCs unmethylated (+ 20 sample analyses characterized as partial assay success, or failure).	Methylation prevalence in groups with different outcomes, Kaplan Meier analysis	Complete response (100% unmeth), Partial response (10% meth, 88% unmeth), Stable disease (8% meth, 84% unmeth), Progressive disease (17% meth, 78% unmeth), no significant differences between groups with different outcomes.
VHL[26]	14 untreated tumor tissues, 14 treated tumor tissues, 2 paired samples as controls (fresh frozen tissues)	Sunitinib	14% of patients methylated at *VHL* region 7896829 before treatment, 64% after treatment with sunitinib (FDR = 0.077, p< 0.001).	t-test (favorable vs poor response)	No significant difference in the extent of *VHL* hypermethylation between those patients who had a favorable vs. poor response to sunitinib (p=0.896).
Multiple genes [29]	102 tumor tissues, 5 normal tissues(fresh frozen tissues)	Sunitinib	At a global methylation level, ccrcc1/ ccrcc4 tumors were more methylated than ccrcc3/ ccrcc2.<br>ccrcc3 tumors had a methylation profile similar to that of NTs. Genes related to polycomb targets (*PRC2, SUZ12, H3K27m3*) were found downregulated by hypermethylation in ccrcc1/4 (hypergeometric test, p < 8e–147), while genes involved in immune response and mitotic cycle in ccrcc4 tumors were upregulated by hypomethylation.	Kaplan Meier curves for ccrcc 1-4, Univariate Cox analyses of OS, PFS.	(Functional status-dependent therapy response)<br>PFS: ccrcc2 (HR= 0.6 [0.34 - 1.1, 95%CI], p=0.08), ccrcc3 (HR= 0.56 [0.24 - 1.3, 95%CI], p=0.2), ccrcc4 (HR= 2.31 [1.1 - 3.3, 95%CI], p=0.02).<br>OS: ccrcc2 (HR= 0.45 [0.25 - 0.8, 95%CI], p=0.007), ccrcc3 (HR= 0.55 [0.24 - 1.3, 95%CI], p=0.2), ccrcc4 (HR= 1.93 [0.97 - 3.8, 95%CI], p=0.06).
SYNPO2 [33]	25 tumor tissues (group1)<br>/ 32 tumor tissues (group2)<br>/ 31 tumor tissues (group 3) (FFPE)	Antiangiogenic agents	The methylation rate in groups 2 and 3 was 21/32(65.6%) and 13/31 (41.9%) respectively.	Stratified univariate models, multivariate Cox regression models, Kaplan -Maier plot.	Myopodin methylation is an independent predictive factor for PFS (HR=0.45 [0.25-0.82, 95%CI], p=0.009), DSS (HR= 0.4 [0.2-0.76, 95%CI], p=0.006), OS (HR= 0.45 [0.25-0.82, 95%CI], p=0.01) for patients under therapy.
Multiple genes [35]	28 tumor tissues (fresh frozen tissues)	Pazopanib	Tumor characterization as $ccrcc_{1-4}$ according to a previous publication from the same authors, with known differences in gene expression and methylation status between subtypes.	Kaplan Meier plot (PFS-OS) compared with log–rank test. Tumor volume reduction compared with ANOVA, Univariate- bivariate Cox regression model	PFS: 9mo, 5mo, 3mo for $ccrcc_{2+3}$, $ccrcc_1$, $ccrcc_4$ (p:0.011). OS: 69mo, 19mo, 5mo for $ccrcc_{2+3}$, $ccrcc_1$, $ccrcc_4$ (p:0.003). Tumor volume decrease: -34%, -6%, -2% for $ccrcc_{2+3}$, $ccrcc_1$, $ccrcc_4$. $ccrcc_{2+3}$ vs. $ccrcc_{1+4}$ significant in bivariate Cox proportional hazard model (p:0.026 for PFS, p:0.04 for OS).
VHL [37]	90 tumor tissues (FFPE)	Sunitinib	*VHL* methylation in 10 of 90 tumor tissues (2/28 for LTRs, 8/62 for other patients).	Fisher exact test	No association of response to sunitinib with *VHL* methylation status (p = 0.718).
PON1 [38]	15 tumor tissues, 15 normal tissues (fresh frozen tissues), TCGA	Sunitinib	Methylation data from TCGA showed hypermethylation of the *PON1* gene in tumor tissues compared to normal tissues.12 of 15 tumor tissues were found hypermethylated in the *PON1* gene.		

	data				
QPCT [39]	14 pairs of RCC tissues from responsive and non-responsive patients for methylation studies. Expression studies on 32 (mRNA) +30 (protein level) +156 (immunocytochemistry) patients with advanced RCC	Sunitinib	Higher methylation in tissues of responders (statistical significance at various CpG regions, only cancer tissues included).	t-test (responders vs. non-responders)<br>PFS (Kaplan- Meier plot)	Greater PFS improvement in sunitinib-treated patients with low *QPCT* expression (p:0.0155) than in the subgroup with high expression (p: 0.4629).
Transposable elements (TE) [40]	24 tumor tissues (FFPE)	PD-1/PD-L1<br><br>inhibitors		Mann Whitney U tests for antiviral gene expression in responders vs. non-responders group	*DDX58, IFIH1*, and *DHX58* expression upregulated in the responder group (13 out of 24, p:0.006, p:0.011, p:0.027).
UQCRH [41]	Prefixed human tissue array	Everolimus	(only immunohistochemistry was performed on tissues).		
CTLA4 [42]	Non-ICB cohort (n=116), ICB cohort (n= 71), TCGA cohort (n= 533) (preservation form not stated)	Immune checkpoint inhibitors	Methylation analysis at two CpG sites<br>Significant hypomethylation in tumor tissues compared to normal adjacent tissues (p<0.001), high degree of comethylation between two CpG sites.	Kaplan-Meier curves, Cox regression for PFS, OS	(analysis performed on ICB cohort)<br>Hypomethylation prolonged PFS (HR= 1.94 [1.09-3.44, 95% CI], p=0.024) and OS (HR= 2.14 [1.01-4.57, 95% CI], p=0.048), statistical significance remained after multivariate analysis.
TCAIM [43]	16 matched tumor-normal adjacent tissues (preservation form not stated)	Sunitinib	Hypermethylation in 7/16 (43,75%) of tumor tissues, no hypermethylation in normal adjacent tissues.		

Abbreviations: RCC: Renal cell cancer; 5-FU: 5-Fluorouracil; FFPE: Formalin-fixed, paraffin-embedded; OS: Overall survival; PFS: Progression-free survival; CI: Confidence interval; mTOR: mammalian Target of Rapamycin; VEGF: Vascular endothelial growth factor; TCGA: The Cancer Genome Atlas Program; AUC: Area under curve; ROC: receiver operating characteristic; HR: Hazard ratio; ORR: overall response rate; FDR: False discovery rate; ccrcc: clear cell renal cell cancer; mo: months; DSS: Disease-free survival; LTR: Long-term responders; ICB: Immune Checkpoint Blockade

Table S4. OHAT rating tool for assessment of risk of bias in mechanistic studies.									
gene (ref)	1. Was administered dose or exposure level adequately randomized?	2. Was allocation to study groups adequately concealed?	3. Were experimental conditions identical across study groups?	4. Were research personnel blinded to the study group during the study?	5. Were outcome data complete without attrition or exclusion from analysis?	6. Can we be confident in the exposure characterization?	7. Can we be confident in the outcome assessment (including blinding of assessors)?	8. Were all measured outcomes reported?	9. Were there no other potential threats to internal validity
ABCG2 [13]	Probably Low	Probably Low	Definitely Low	Probably Low	Probably Low	Definitely Low	Probably Low	Definitely Low	Probably Low
RASSF1 [14]	Probably Low	Probably Low	Definitely Low	Probably Low	Probably High	Definitely Low	Probably Low	Probably High	Probably Low
XAF1 [15]	Probably Low	Probably Low	Definitely Low	Probably Low	Probably High	Probably Low	Probably Low	Probably High	Probably High
32 promoter CpG islands [17]	Probably Low	Probably Low	Definitely Low	Probably Low	Probably Low	Probably Low	Probably Low	Probably Low	Probably Low
Cx32 [18]	Probably Low	Probably Low	Definitely Low	Probably Low	Probably Low	Probably Low	Probably Low	Probably Low	Probably Low
MSH2 [24]	Probably Low	Probably Low	Definitely Low	Probably Low	Definitely Low	Definitely Low	Probably Low	Definitely Low	Probably Low
SLFN11 [28]	Probably Low	Probably Low	Definitely Low	Probably Low	Probably Low	Probably Low	Probably Low	Definitely Low	Probably Low
XAF1 [16]	Definitely High	Probably Low	Definitely Low	Probably Low	Probably High	Probably Low	Probably Low	Probably High	Probably High
NEFH [19]	Probably Low	Probably Low	Definitely Low	Probably Low	Probably Low	Probably Low	Probably Low	Probably Low	Probably Low
FLT1, KDR [25]	Probably Low	Probably Low	Definitely Low	Probably Low	Definitely Low	Probably Low	Probably Low	Definitely Low	Probably Low
ASC/TMS1 [27]	Probably High	Probably Low	Definitely Low	Probably Low	Probably Low	Probably Low	Probably Low	Probably Low	Probably Low
OCT2 [30]	Probably Low	Probably Low	Definitely Low	Probably Low	Definitely Low	Probably Low	Probably Low	Definitely Low	Probably Low
DAB2IP [31]	Probably High	Probably Low	Definitely Low	Probably Low	Probably Low	Probably Low	Probably Low	Probably Low	Probably Low
Multiple pharmacogenes [32]	Probably High	Probably Low	Definitely Low	Probably Low	Probably Low	Probably Low	Probably Low	Definitely Low	Probably Low
ASPP1 [34]	Probably High	Probably Low	Definitely Low	Probably Low	Probably Low	Probably Low	Probably Low	Probably Low	Probably Low
LIFR [36]	Probably High	Probably Low	Definitely Low	Probably Low	Probably Low	Probably Low	Probably Low	Probably Low	Probably Low
DCLK1 [21]	Probably Low	Probably Low	Probably Low	Probably Low	Probably Low	Not reported	Probably Low	Probably Low	Probably Low
PON1 [38]	Probably High	Probably Low	Definitely Low	Probably Low	Probably Low	Probably Low	Probably Low	Probably Low	Probably Low
QPCT [39]	Probably Low	Probably Low	Definitely Low	Probably Low	Probably Low	Definitely Low	Probably Low	Probably Low	Probably Low
TE [40]	Probably Low	Probably Low	Definitely Low	Probably Low	Probably Low	Definitely Low	Probably Low	Probably Low	Probably Low
UQCRH [41]	Probably Low	Probably Low	Definitely Low	Probably Low	Probably Low	Probably Low	Probably Low	Probably Low	Probably Low
TCAIM [43]	Probably Low	Probably Low	Definitely Low	Probably Low	Probably Low	Probably Low	Probably Low	Probably Low	Probably Low

Scale	Definitely Low	Probably Low	Probably High or Not reported	Definitely High

Table S5. Newcastle – Ottawa scale for assessing risk of bias in observational studies.									
Gene (ref)	Newcastle Ottawa scale								Study design
	Selection				Comparability	Outcome/exposure			
XAF1 [16]	*		*	* *	* *	* *			cross sectional
NEFH [19]		*	*	*		*	*	*	cohort
FLT1, KDR [25]		*	*	*	*	*	*		cohort
ASC/TMS1 [27]	*		*	* *	*	* *			cross sectional
OCT2 [30]	*		*	* *	* *	* *	*		cross sectional
DAB2IP [31]	*	*	*	*	* *	*	*	*	cohort
Multiple pharmacogenes [32]			*	* *	* *	* *	*		cross sectional
ASPP1 [34]			*	* *	* *	* *	*		cross sectional
LIFR [36]			*	* *	* *	* *	*		cross sectional
DCLK1 [21]	*		*	* *		* *	*		cross sectional
VHL [20]	*	*	*	*		*	*	*	cohort
CST6, LAD1 [22]	*	*	*	*		*	*	*	cohort
VHL [23]	*	*	*	*		*	*		cohort
VHL [26]		*	*	*		*	*	*	case control
Multiple genes [29]	*	*	*	*		*	*	*	cohort
SYNPO2 [33]	*	*	*	*	* *	*	*		cohort
Multiple genes [35]		*	*	*	*	*	*	*	cohort
VHL [37]	*	*	*	*		*	*	*	case control
PON1 [38]	*		*	* *	* *	*			cross sectional
QPCT [39]		*	*	*		*	*	*	cohort
Transposable elements [40]		*	*	*		*	*	*	cohort
UQCRH [41]			*	*	* *	* *			cross sectional
CTLA4 [42]	*	*	*	*	*	*	*	*	cohort
TCAIM [43]			*	*	* *	* *			cross sectional

Table S6. Origin of the cell lines used for in vitro analyses in the included studies of the review							
Metastatic sites (originated from)	Primary clear cell RCC			Primary papilllary RCC	Primary chromophobe RCC	Other RCC subcategories	Not stated
ACHN (papillary RCC)	UOK121	UOK115	SNU228	UOK112		SN12C	UO-31
SK-RC-45 (clear cell RCC)	UOK143	UOK122	SNU333	SNU482		TK-10	WWC1
SK-RC-29 (clear cell RCC)	UOK181	UOK123	SNU349				KETR-3
CAKI-1 (clear cell RCC)	A-498	UOK124	SNU267				
SUT002 (clear cell RCC)	786-O	UOK130	769-P				
SOR001 (clear cell RCC)	RFX-393	RCC-MF	CCF-RC1				
SKRC39 (papillary RCC)	UOK101	RCC-HS	OS-RC-2				
	UOK105	RCC-GS	UMRC2				
	UOK108	A-704	OS5K-1				
	UOK109	CAKI-2	OS5K-2				
	UOK110	SNU1272	OS5K-3				

Abbreviations: RCC, Renal cell cancer

Table S7. Histological categorization of the analyzed tissue samples of the included studies					
Gene/ reference	Total cases	Clear cell RCC	Papillary RCC	Chromophobe RCC	Other histologies
XAF [16]	20 (fresh frozen tissues)	Not Stated			
NEFH [19]	114 (fresh frozen tissues)	82	24	0	8
	18 (FFPE)	16	1	1	0
FLT1,KDR [25]	8 (fresh frozen tissues)	Not stated, methylation analysis on TCGA-KIRC data			
	13 (FFPE)	13	0	0	0
ASC/TMS1 [27]	202 (fresh frozen tissues)	185	9	8	0
OCT2 [30]	46 (fresh frozen tissues)	38	4	0	4
	31 (TMA)	10	11	10	0
DAB2IP [31]	439 (TMA)	333	57	49	0
Multiple genes +OCT2 [32]	34 (primary tumor)	34	0	0	0
	17 (metastases)	17	0	0	0
ASPP1 [34]	20 (fresh frozen tissues)	20	0	0	0
	94 (TMA)	86	3	0	5
LIFR [36]	25 (FFPE)	25	0	0	0
DCLK1 [21]	172 (TMA)	Not stated, methylation analysis on TCGA-KIRC data			
VHL [20]	78 (FFPE)	78	0	0	0
CST6, LAD1 [22]	18 (FFPE)	16	1	1	0
VHL [23]	143 (FFPE)	143	0	0	0
VHL [26]	28 (fresh frozen tissues)	28	0	0	0
Multiple genes [29]	102 (fresh frozen tissues)	102	0	0	0
SYNPO2 [33]	25 (FFPE)	20	3	2	0
	32 (FFPE)	28	2	1	1
	31 (FFPE)	25	3	0	2
Multiple genes [35]	28 (fresh frozen tissues)	28	0	0	0
VHL [37]	90 (FFPE)	90	0	0	0
PON1 [38]	25 (fresh frozen tissues)	Not stated, methylation analysis on TCGA-KIRP data			
QPCT [39]	28 (methylation analyses)	28	0	0	0
	32 (mRNA expression analyses)	32	0	0	0
	30 (protein expression analyses)	30	0	0	0
	156 (immunocytochemistry)	156	0	0	0
Transposable elements [40]	24 (FFPE)	24	0	0	0
UQCRH [41]	TMA	Not stated, survival analysis on TCGA-KIRC data			
CTLA4 [42]	116	116	0	0	0
	71	67	4		
TCAIM [43]	16	Not stated			
Abbreviations: RCC, Renal cell cancer; FFPE, Formalin-fixed, paraffin-embedded; TMA, Tissue Microarray; TCGA, The Cancer Genome Atlas Program; KIRC, Kidney renal clear cell carcinoma; KIRP, Kidney renal papillary cell carcinoma					
